# Incorporating Objective Behavioral and Biological Measures in a Large-Scale Decentralized Mobile Health Trial for Depression: Randomized Controlled Trial

**DOI:** 10.2196/92589

**Published:** 2026-10-09

**Authors:** Wendy S-Y Lau, Caroline M Swords, Margaret W Thairu, Nelson Hidalgo, Raquel Tatar, Hadley Rahrig, Christine D Wilson-Mendenhall, Daniel W Grupe, Robin I Goldman, Nathaniel J Vack, Craig Ferguson, Gabriela Valdivia, Kris Sankaran, Corrina Frye, Cortland J Dahl, Robert Lewis, Estelle T Higgins, Mason Garza, Zishan Jiwani, Matthew J Hirshberg, Amit Bernstein, Ellen Converse, Sona Dimidjian, Earlise C Ward, Gregory E Miller, Thomas W McDade, Rosalind W Picard, Jo Handelsman, Melissa A Rosenkranz, Heather C Abercrombie, Richard J Davidson, Simon B Goldberg

**Affiliations:** 1 Center for Healthy Minds, University of Wisconsin–Madison, Madison, WI, United States; 2 Department of Counseling Psychology, University of Wisconsin–Madison, 335 Education Building, 1000 Bascom Mall, Madison, WI, 53703, United States, 1 (608) 265-8986; 3 Wisconsin Institute of Discovery, University of Wisconsin–Madison, Madison, WI, United States; 4 MIT Media Lab, Massachusetts Institute of Technology, Cambridge, MA, United States; 5 Humin, Madison, WI, United States; 6 Department of Statistics, University of Wisconsin–Madison, Madison, WI, United States; 7 Department of Psychology, University of Wisconsin–Madison, Madison, WI, United States; 8 Department of Biomedical Engineering, University of Wisconsin–Madison, Madison, WI, United States; 9 School of Psychological Science, University of Haifa, Haifa, Israel; 10 PAU-Stanford PsyD Consortium, Palo Alto University, Palo Alto, CA, United States; 11 Department of Psychology and Neuroscience, Crown Institute, University of Colorado Boulder, Boulder, CO, United States; 12 Department of Family Medicine and Community Health, School of Medicine and Public Health, University of Wisconsin–Madison, Madison, WI, United States; 13 Department of Psychology, Institute for Policy Research, Northwestern University, Evanston, IL, United States; 14 Department of Anthropology, Northwestern University, Evanston, IL, United States; 15 Department of Plant Pathology, University of Wisconsin–Madison, Madison, WI, United States; 16 Department of Psychiatry, School of Medicine and Public Health, University of Wisconsin–Madison, Madison, WI, United States

**Keywords:** digital health, depression, well-being, biological measurement, behavioral tasks, decentralized clinical trial

## Abstract

**Background:**

Decentralized (ie, fully remote) clinical trials (DCTs) offer a scalable and ecologically valid approach to evaluating digital mental health interventions (DMHIs). Yet, incorporating objective measures of well-being within DCTs poses novel methodological and feasibility challenges that can limit the rigor and precision of data collection in the field.

**Objective:**

We recently conducted the Behavior, Biology, and Well-being (BeWell) Study, a large-scale, 3-arm randomized DCT evaluating the Healthy Minds Program app (Humin)—a self-guided DMHI that includes didactic well-being information and meditation components. The current paper’s objective is to describe the methodological approach to collecting and processing a multimodal battery of psychological (questionnaires and structured clinical interview), biological (blood and stool assays), and behavioral (stimulus-elicited affect [SEA] task, video log, and Mnemonic Similarity Task [MST]) measures, longitudinally across baseline, the 4-week intervention period, and 17-week follow-up. Secondarily, we evaluate its feasibility.

**Methods:**

The BeWell Study methodology is described. Descriptive statistics characterized recruitment and retention, as well as data completion and data quality. Binary logistic regressions evaluated whether recruitment sources, demographics, and depression severity predicted measure completion and data quality.

**Results:**

From June 1, 2022, through July 15, 2024, 52,052 individuals started the screening questionnaire. Of these, 2.22% (n=1156 participants; mean age 38.69, SD 12.14 years; women=75.50%; non-Hispanic White=68.88%) completed the screener, met eligibility criteria (including elevated depressive symptoms [Patient Health Questionnaire-9 [PHQ-9] score≥5]), completed baseline measures, were enrolled, and were randomized. Participants were recruited from Craigslist (694/1154, 60.14%), a US-based advertisement website, and university email messages (107/1154, 9.27%); participants were enrolled from every US state. Across the study, 84% (971/1156) of participants were retained (completed ≥1 measure at 17-week follow-up). Across time points, ≥81.40% (941/1156) of participants completed questionnaires and ≥75.17% (869/1156) completed biological samples. Of those completing questionnaires, ≥84.14% (796/946) completed SEA and video log tasks and ≥91.75% (868/946) completed the MST. Of completed assessments, high-quality or analyzable data rates across all time points were ≥97.89% (926/946) for questionnaires, ≥90.20% (773/857) for SEA, ≥84.60% (725/857) for video logs, ≥94.20% (1056/1121) for the MST, ≥98.84% (849/859) for dried blood spots, and ≥99.43% (865/870) for microbiome samples. Greater depressive symptoms at screening, but not age, predicted completion of baseline measures (odds ratios 0.96‐0.99; *P*<.001). For enrolled participants, recruitment sources and baseline depressive symptoms were not predictive of data completion or quality. Older age and having a college degree predicted significantly higher rates of data completion across all data types (questionnaire, behavioral, and biological) at week 4 or week 17 follow-ups.

**Conclusions:**

This methodological approach offers one model of incorporating objective measures of well-being into DCTs testing DMHIs. Implications and limitations of our approach are discussed.

## Introduction

Large-scale clinical trials have historically been conducted in person. Yet, in-person centralized data collection is limited by geographical restrictions on participant recruitment, high operational costs associated with large study teams and specialized facilities, and the complex logistics of multisite coordination. Although the COVID-19 pandemic significantly disrupted in-person clinical trials, it simultaneously accelerated the adoption of novel digital research methods compatible with COVID-19–related safety concerns and restrictions surrounding in-person clinical research [1,2]. One such example is decentralized clinical trials (DCTs), which apply digital technologies to conduct and monitor remote study activities and are less location-restricted; thus, DCTs offer a novel approach to overcoming challenges associated with in-person clinical trials.

In addition to overcoming the challenges of in-person trials, DCTs are particularly well-suited to testing digital mental health interventions (DMHIs), which are technology-based interventions designed to improve mental health that can be delivered via tools such as smartphones. Like DCTs, DMHIs are not bound by geographic restrictions, making them well-suited for scalable and remote evaluation. DMHIs hold promise for addressing numerous psychiatric conditions, including depression, substance misuse, anxiety, and trauma-related disorders [3-5]. Given that the need for mental health treatment has outpaced human resources, DMHIs offer practical, real-world benefits, including the ability to deliver evidence-based interventions at scale while maintaining anonymity, engagement, and flexibility [5,6]. Yet, even as DMHIs make it possible to reach larger and more geographically dispersed samples, researchers face novel and substantial methodological challenges associated with remotely evaluating these interventions with the same rigor as large-scale, in-person clinical trials. Meeting this challenge requires methods that not only assess clinical outcomes but also incorporate objective behavioral and biological markers to clarify the mechanistic effects of DMHIs on mental health in ecologically valid contexts.

In this regard, DCTs offer both methodological advantages and unique challenges for mechanistic mental health research. In terms of advantages, they may be particularly well-suited for multimodal sampling of potentially relevant biomarkers across interacting psychological and physiological systems in ecologically valid settings [7-9]. For example, the remote collection of cardiovascular biomarkers, measured via wearable sensors (eg, biometric vests and wrist-worn devices), and the ambulatory assessment of psychological states and behavior have been increasingly integrated into DCTs [7,10]. Yet, very few trials have included remotely collected biological samples (eg, blood and microbiome), which are commonly collected in the laboratory but can, in theory, be self-sampled in DCTs. Challenges to blood and microbiome sample collection—2 biomarkers with particular mental health relevance [11,12]—include the burden on participants to collect sufficient samples and the burden on study teams to ensure samples are temperature-controlled, which is required for some sample collection methods. Similarly, although some digital health trials have included objective behavioral markers of mental health [13], these measures are not widely implemented, in part because of the complex methodological challenges involved in collecting and processing video and audio data.

This gap in the multimodal collection of biological and behavioral data is important. Biological measures are necessary to assess key inflammatory and microbial markers implicated in the neuroimmune and brain-gut-microbiota axes [11,12]. These axes may be especially relevant for mental health interventions as inflammatory signals and gut microbiota directly and indirectly modulate the central nervous system, with consequences for stress, resilience, and mental health [11,12,14,15]. Similarly, past research demonstrates that behavioral measures, such as natural language use [16], reward sensitivity [17], and performance on cognitive tasks [18,19], are meaningful markers of well-being.

Multimodal assessment and the inclusion of objective measures of well-being may be particularly relevant for DCTs testing DMHIs for depression. Depression, a leading cause of disability worldwide [20], is influenced by a complex combination of psychological, behavioral, and biological factors. For example, past research demonstrates depression is associated with disruptions in natural language (eg, greater first-person pronoun use), disruptions to reward sensitivity [17], cognitive disruptions [19], and elevated levels of inflammation [21]. Given the public health burden associated with depression [20], scalable and accessible interventions that are rigorously tested within DCTs, and which allow for the collection of psychological, behavioral, and biological measures to understand how DMHIs are, or are not, improving depressive symptoms and objective measures, are urgently needed [22]. Yet, methodological challenges remain for researchers aiming to remotely incorporate a multimodal battery of measures that can rigorously examine the effects of DMHIs across these domains in large-scale DCTs. Furthermore, researchers also face challenges in using methods that are feasible and not overly complicated or burdensome for participants to complete. Although prior studies have examined remote digital measures or biomarkers in mental health research generally, approaches to incorporating multimodal batteries that include such measures within large-scale DCTs of DMHIs are not well established or widely implemented.

Accordingly, through a National Institutes of Health–funded multidisciplinary network, we developed a methodology for a DCT to remotely deliver a smartphone-based DMHI—the Healthy Minds Program (HMP) app (Humin)—to a large, national sample of adults selected for clinically elevated depressive symptoms. As part of the Behavior, Biology, and Well-being (BeWell) Study, we developed a methodology to integrate psychological (eg, structured clinical interviews and self-report questionnaires), behavioral (eg, video ecological momentary assessment [EMA] data and Mnemonic Similarity Task [MST]), and biological (eg, blood and microbiome) measures into this 3-armed randomized DCT. This paper is not intended to report on the efficacy of the HMP app or the trial’s primary clinical outcomes. Rather, this paper presents the methodology used in the BeWell Study to remotely collect and process a multimodal battery of measures for researchers seeking to implement multimodal assessments within DCTs—particularly for testing DMHIs. Secondarily, we aim to assess the feasibility of the developed methodology by examining data completion and quality, as well as predictors (ie, recruitment source, demographics, and depressive symptom severity) of data completion and quality across the study. We hope that these methodological details and the evaluation of the feasibility of this approach will be useful to other researchers seeking to implement multimodal assessment within large-scale DCTs, particularly those testing DMHIs.

## Methods

### Study Design

This study was a prospectively registered (ClinicalTrials.gov registration: NCT05183867) 3-arm, longitudinal DCT testing the HMP app [23]. It was funded by grants from the National Institutes of Health, Hope for Depression Research Foundation, and the Brain & Behavior Research Foundation. Participants were recruited remotely via advertisements listed on clinicaltrials.gov, Craigslist, ResearchMatch, WithPower, social media platforms (Facebook [Meta Platforms, Inc], Twitter [Twitter, Inc], subsequently rebranded X, Instagram [Instagram, LLC], and Reddit [Reddit, Inc]), and web postings from the National Alliance on Mental Illness (NAMI), Mental Health America, the Center for Healthy Minds, and Madison 365. Email advertisements were sent to students and staff at the University of Wisconsin (UW)–Madison and those in the Center for Healthy Minds Participant Registry. Flyers were posted locally in the Madison, WI area and were mailed to local and national mental health clinics and organizations (eg, NAMI and Mental Health America). Interested participants were directed to a landing page within the REDCap software (Vanderbilt University) [24,25], a HIPAA (Health Insurance Portability and Accountability Act)-compliant web app designed to support data collection and management for research studies. The BeWell Study landing page included a video of the study’s principal investigator describing the study and a virtual consent form. All participants provided informed consent and completed a baseline assessment, which took approximately 60‐90 minutes.

Study measures and their timeline are depicted in Figure 1. At baseline, participants completed questionnaires (45‐60 minutes) and behavioral tasks. Behavioral tasks included the MST [18,26], a hippocampal-dependent measure of episodic memory delivered via smartphone, and 2 video-based tasks that collected video EMA data through an app made specifically for the study called the Teddy app (MIT Media Lab; ie, stimulus-elicited affect [SEA] task and video log, described further below).

**Figure 1.**
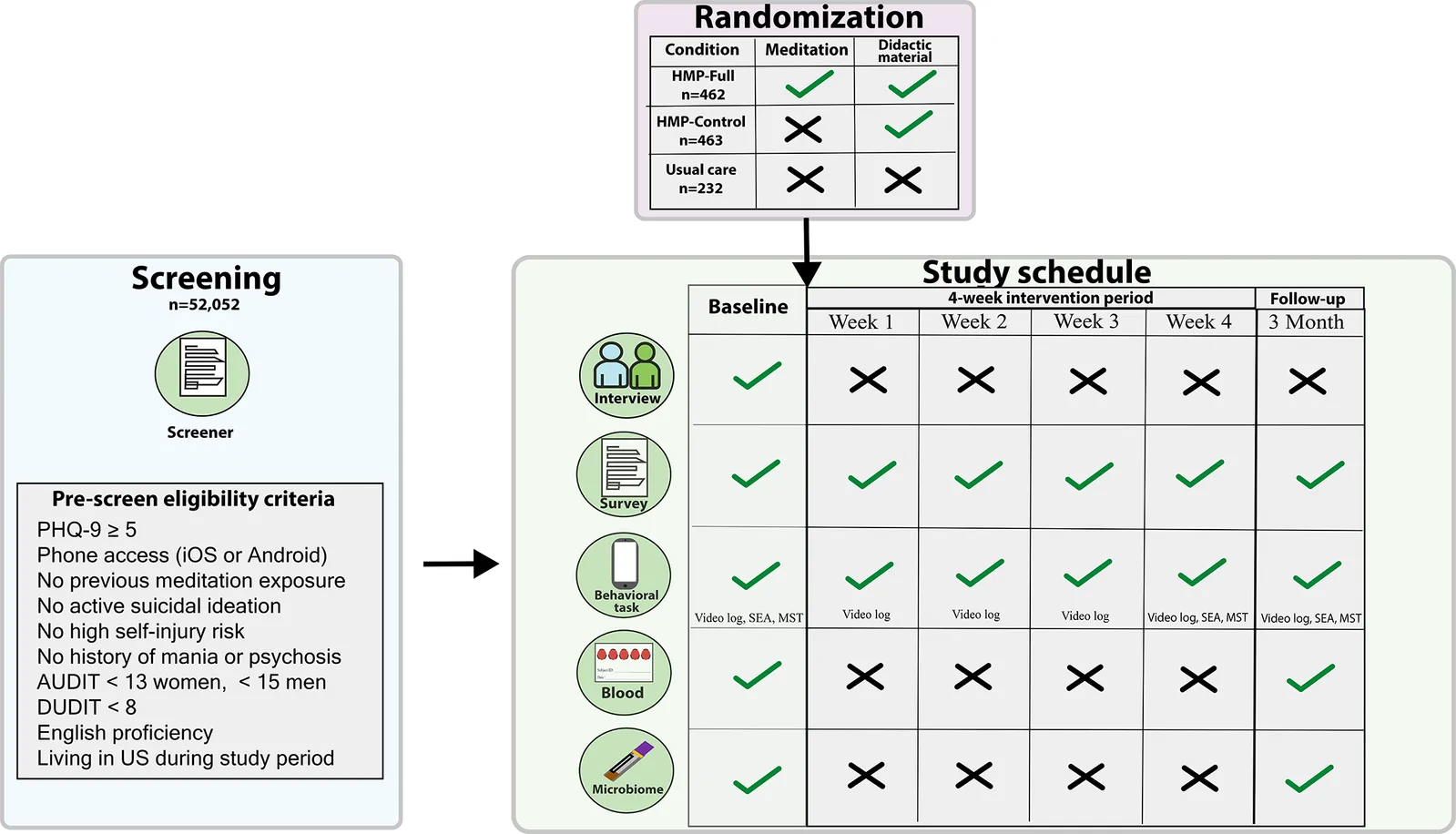
Experimental overview. This figure illustrates the experimental overview of the Behavior, Biology, and Well-being (BeWell) Study and illustrates the process of screening, enrollment, randomization, and measures completed by participants during the 4-week intervention period and 17-week follow-up. Prior to randomization, participants were required to complete prescreen eligibility criteria and baseline measures. HMP-Full: Healthy Minds Program app with full content (meditation and didactic material); HMP-Psychoed: Healthy Minds Program app with didactic material only; usual care: condition in which participants were not assigned to any digital interventions. MST: Mnemonic Similarity Task; SEA: stimulus-elicited affect.

Participants determined to be eligible were then mailed baseline biological sample collection kits and a reloadable debit card (ie, Focus Blue) issued by US Bank. Automated REDCap emails invited participants to complete a remote clinical interview (conducted over a HIPAA-compliant video platform) and return their biological samples (dried blood spot [DBS] cards and stool samples). Participants who completed these baseline measures were enrolled and randomized via REDCap’s randomization module to 1 of the 3 study groups in a 2:2:1 ratio: HMP-Full (Healthy Minds Program app with full content), HMP-Psychoed (Healthy Minds Program app with didactic material only), or usual care (described more fully below). Participants assigned to one of the HMP conditions (HMP-Full and HMP-Psychoed) were encouraged to use the app daily during the 4-week intervention period.

Each week of the 4-week intervention period, participants completed weekly online surveys sent via email automatically with REDCap. Upon completing the weekly surveys, participants were directed to the Teddy app to complete video EMA tasks. Participants were given 1 week to complete each weekly assessment. At posttreatment (ie, week 4), participants completed questionnaires, both video EMA tasks collected through the Teddy app—SEA task and video logs—and the MST. This same battery was administered at the 17-week follow-up, along with a second collection of biological measures (ie, blood and stool). Participants were compensated up to US $265 through their Focus Blue card or by check, at 3 time points (baseline, post treatment, and 17-week follow-up). Participants who completed all measures or attempted all measures but could not complete them due to technical or shipping errors received a US $50 bonus. For more compensation details, see Table S1 in Multimedia Appendix 1. Data collection began on June 1, 2022, and was completed on July 15, 2024, with direct support from 2.5 full-time staff.

### Participants

Inclusion criteria were: age 18 to 65 years, elevated Patient Health Questionnaire-9 (PHQ-9) score of ≥5 [27] at screening, access to a smartphone (iOS or Android), and English proficiency. Exclusion criteria were: significant meditation experience (ie, daily meditation practice in the past 6 months, weekly meditation in the past year, attendance of retreat with meditation component, and/or previous use of HMP app); current suicidal intent based on clinical interview; self-reported history of psychosis and/or mania; Alcohol Use Disorders Identification Test (AUDIT) [28] score of ≥15 for men, ≥13 for all other genders; Drug Use Disorders Identification Test (DUDIT) [29] score of ≥8; or living outside the United States. Prior to enrollment, participants were also required to complete a remote clinical interview, submit at least one of 2 biological samples (DBS or microbiome), complete baseline questionnaires, and attempt (ie, opened but not completed due to technical error) the behavioral tasks (video EMA and MST).

### Measures and Materials

#### Digital Intervention

The HMP app is a free, publicly available, self-guided, DMHI. Each module is dedicated to 1 of 4 pillars of well-being: awareness, connection, insight, and purpose. Awareness includes capacities such as meta-awareness and attention regulation. Connection includes capacities such as kindness and appreciation directed toward oneself and others. Insight includes capacities such as self-inquiry and the ability to examine how internal experiences such as thoughts and emotional reactions shape perception. Purpose includes the ability to connect one’s daily life experiences with one’s values and motivations. The HMP app includes psychoeducation and >100 guided meditation practices corresponding to these pillars of well-being [3,30,31]. The content of the HMP app used in the BeWell Study was nearly identical to the publicly available version, with modifications made to introductory content to frame the program around mental health promotion. The layout of the app’s main page was also modified to clarify which elements were part of the 4-week intervention period. The active-control version of the HMP app (HMP-Psychoed)—included to isolate the added contribution of meditation beyond didactic well-being content—was modified to contain only psychoeducation, with guided meditation practices removed. Those in usual care received no study-specific intervention but were invited to access the public version of the HMP app after the study concluded.

#### Remote Clinical Interview

To fully assess the criteria for *DSM-5* (*Diagnostic and Statistical Manual of Mental Disorders *[Fifth Edition]) depressive disorders, a brief clinical diagnostic interview adapted from the depression module of the Structured Clinical Interview for *DSM-5* Research Version (SCID-5-RV) [32] was conducted remotely over a HIPAA-compliant video platform.

#### Questionnaires

Standardized questionnaires assessed self-reported levels of psychological symptoms, well-being, and physical health. A complete list of measures administered at each time point is described in the study’s registration on ClinicalTrials.gov (identifier: NCT05183867). Additionally, a complete list of questionnaires at each time point is included in Table S2 in Multimedia Appendix 1.

#### Behavioral Data

##### Video EMA

Video EMA data (ie, SEA task and video logs) were collected with the Teddy app—an app created for the BeWell Study that is both Android and iOS compatible. The Teddy app is open-source, with the version used in BeWell and an updated version publicly available [33]. While in the app, participants were greeted and guided through video EMA tasks by Teddy, a teddy bear avatar. For the SEA task, participants approved turning on their phone camera to capture their facial expressions and viewed an approximately 50‐ to 60-second humorous baby-themed video and rated the video’s humor on a scale from 0 to 3. For the video log task, participants left their camera on and were asked to create a video log describing a pleasant or unpleasant experience they had experienced in the past 24 hours. Afterward, participants chose 1 of 3 options to say goodbye to Teddy, ranging from enthusiastic or positive (“See you later!!!” or “Thanks for the video!”) to neutral (“Bye.”). An illustration of the Teddy app can be found in Figure 2. Although Teddy has not been independently validated, the linguistic and facial features extracted from Teddy have been associated with depressive symptoms [34,35].

**Figure 2.**
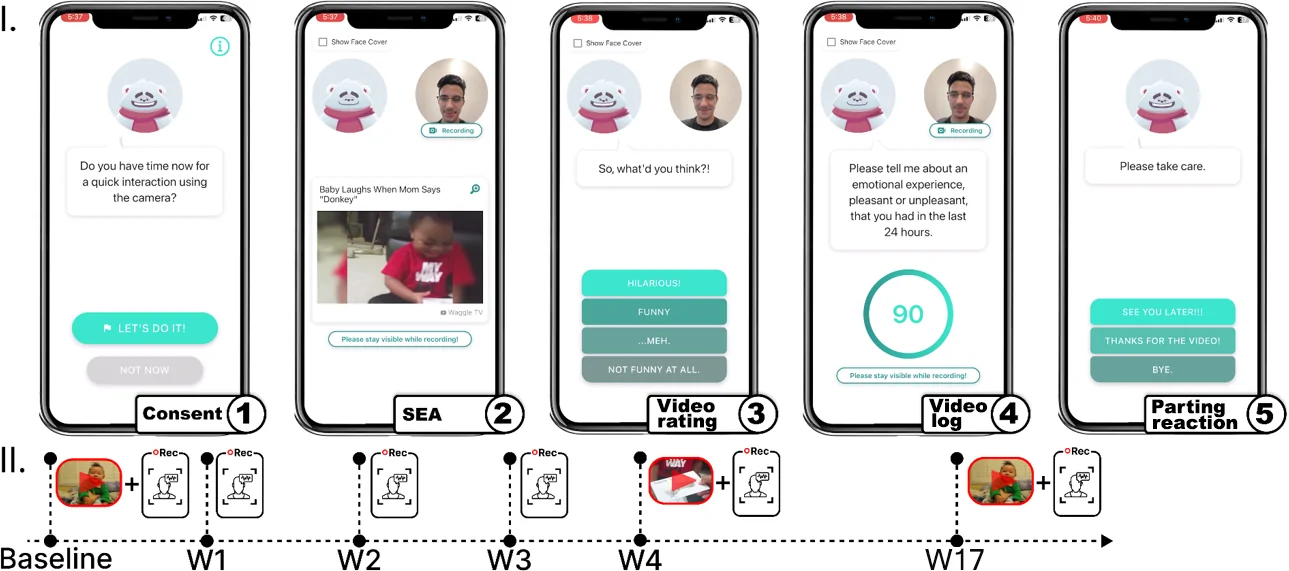
Teddy app. Diagram of (I) user experience interaction with the Teddy research platform, including the facial measurement interactions, “Stimulus Elicited Affect” (SEA) task and “Video log,” and (II) the timeline of the study, where “W1-4” denotes week 1 to week 4 time points, with “Video Logs” done at every time point and “Internet videos” assigned at preintervention, week 1, and week 17 follow-up. The example participant included in this figure was not an actual participant. Due to a technical error, some participants watched SEA videos at random in weeks 1, 2, and 3.

##### MST

The MST [18,26,36] is a hippocampal-dependent measure of episodic memory. This task was adapted by Grupe et al [18] for delivery via smartphone within a remotely recruited adult sample. In this 320-trial task, participants are shown a series of objects, which they categorize as “new,” “old,” or “similar” based on objects that they have been previously shown. Each stimulus is displayed for 10 seconds, followed by a 0.5-second interstimulus interval. The MST has demonstrated acceptable test-retest reliability and construct validity (ie, differentiating age groups; [26]). For more information on the adapted MST, see the study by Grupe et al [18].

### Biological Samples

#### DBS Samples

DBS samples—capillary blood dried on specialized filter paper following a finger stick with a sterile lancet—were collected to measure inflammatory proteins (C-reactive protein [CRP], interleukin [IL]-6, IL-10, and tumor necrosis factor α [TNF-α]) [36,37]. To collect DBS samples, the following materials were provided to participants: a sterile pad, 2 alcohol preps, 2 disposable lancets, 2 Whatman #903 protein saver cards, 2 gauze pads, 2 bandages, a plastic sleeve, and a desiccant pack (see Table S3 in Multimedia Appendix 1).

#### Microbiome

Stool samples were collected to characterize participants’ gut microbiomes using 2 DNA-based methods: 16S rRNA gene amplicon sequencing and whole-genome sequencing (WGS). These complementary methods enable comprehensive profiling of the microbial diversity present in the gut. Specifically, sequences of 16S rRNA gene amplicons provide taxonomic information, identifying the types of bacteria present, whereas WGS offers insights into the functional potential of the microbiome by analyzing the complete metagenomic content. Instructions for sample collection provided to participants can be found in Table S4 of Multimedia Appendix 1. Microbiome samples were collected with the OMNIgene•GUT kit [38], which included an OM-ACI toilet attachment, sample tube, spatula, spoon for fecal samples, specimen collection device, gloves, and a biohazard specimen bag.

### Data Collection and Processing

#### Digital Intervention

Participants were emailed a link through REDCap, which allowed them to download the DMHI (HMP-Full or HMP-Psychoed) onto their device and simultaneously linked their HMP usage with their REDCap participant ID. Automated email reminders were sent through REDCap to those who did not download the HMP app within a week after randomization. The reminder email included a link to a step-by-step instructional video to guide the participants through the process. The following usage data were recorded through the HMP app: date, time, duration, device type, psychoeducation lesson or meditation practice, week, psychoeducation lesson category (awareness, connection, insight, or purpose), and name of specific type of psychoeducation lesson or meditation practice.

#### Clinical Interview

Clinical interview data were collected by a team of licensed mental health professionals or supervised trainees from clinically oriented graduate programs (counseling psychology, clinical psychology, or school psychology). Interviewer availability was shared via Google Calendar with time zone–specific links. Participants emailed to identify possible appointment times and were assigned to interviewers by the study staff; which were conducted virtually via a HIPAA-compliant platform (Webex; Cisco Systems, Inc) [39]. In cases of technical difficulties, interviews were conducted by phone. Clinical interviews took approximately 1 hour. Deidentified documentation of the interview and research diagnoses of depressive disorders was captured within REDCap. Licensed psychologists and a licensed marriage and family therapist supervised interviewers in weekly group meetings to guide diagnostic specificity and documentation.

#### Questionnaires

To prevent participants from entering the study multiple times, we used a Python script to compare newly enrolled participants’ email addresses to existing participants. Potential duplicates were also flagged by computing the normalized Levenshtein distance on names, addresses, and phone numbers, which quantifies similarity by counting the minimum edits needed for a match. Records with a Levenshtein distance below 0.30 were reviewed. When duplication was suspected, only the first case was included, and subsequent attempts were removed. Surveys were administered through REDCap. Questionnaires in which participants passed an attention check (ie, “I have been randomly selecting responses on this survey”) were deemed high quality.

#### Behavioral Measures

##### MST

A link to complete the MST via smartphone was embedded in REDCap surveys. Once the data were collected, outliers were identified based on the criteria used in a previous study [18]. Specifically, individuals with missing behavioral responses for >25% of trials and scores >3 SD below the mean and clearly distinct from the sample distribution were considered low quality.

##### Video EMA

Participants downloaded the Teddy app from an individualized link emailed to them at baseline via REDCap and used it to complete both video EMA tasks (SEA task and video logs) at the scheduled assessment time points. After completing self-report questionnaires in REDCap, participants were directed to the Teddy app, which transferred their participant ID number across platforms. Due to a technical error between Teddy and REDCap, some participants were randomly assigned additional SEA tasks in weeks 1 to 3. Participants provided consent to have their video and audio recorded. Video data submitted by participants were encrypted before transit and were stored encrypted on the servers. A select number of study staff assisting with study coordination were able to access a portal to determine whether video EMA tasks attempted in Teddy were marked as attempted and/or completed by participants. Participants were instructed to reach out to the study team should they have doubts about whether the data had been uploaded. Additionally, an API was built to transfer data quality assessments made by the team monitoring Teddy data into REDCap.

The videos collected using Teddy contain visual and acoustic information. To assess the quality of visual data, we evaluated facial luminance, facial contrast, and interocular head scale, following the methodology used by Hoque et al [40]. Facial action units were extracted using Affdex 2.0 (Affectiva, Inc) [41], a proprietary software shown to perform fairly across diverse demographics based on both mobile and computer settings. We analyzed face-tracking confidence and head-pose features from Affdex 2.0 as helpful metrics of feature extraction reliability. An overview of these visual quality metrics is presented in Figure S1 and Table S6 in Multimedia Appendix 1. Furthermore, sessions were determined to be of low quality if any of the following criteria averaged across the session were met: (1) face tracking confidence was below the 5th percentile, (2) facial luminance was below 40 on a 0‐255 grayscale, (3) head scale exceeded 3.3 interocular distance, or (4) attention measure was below the 5th percentile. The thresholds for luminance and head scale were based on visual inspection, which showed that lower luminance resulted in excessively dark frames and higher head scale indicated participants were too close to the camera.

Only the video log included participants’ speech. The quality of this acoustic speech data was assessed using voice activity detection (VAD) via Silero (Silero Team) [42] and transcript validity using WhisperX (Bain) [43]. Sessions were determined to be low quality if they contained no interpretable transcript (as extracted from WhisperX) or if participants spoke for less than 10% of the 90-second recording, as measured by VAD. A visualization illustrating the quality of video EMA samples is shown in Figure S1 and Table S6 in Multimedia Appendix 1.

### Biological Samples

#### Overview

Participants were shipped a biological sample collection kit, assembled by the study team, that included materials for both blood and microbiome sample collection via FedEx. Detailed instructions were provided in written and video format for self-collection of both biological sample types (see Tables S3-S5 in Multimedia Appendix 1). Participants were also given the option of scheduling a video call with 1 of the 3 (2 full-time and one half-time) study staff for assistance. They were instructed to return all biological samples via FedEx at a physical FedEx location or by scheduling a FedEx pickup from their residence (Table S5 in Multimedia Appendix 1). The study team arranged FedEx pickups upon participant request.

#### DBS Sample

Participants were instructed to place the provided materials on the collection pad. They cleaned their middle or ring finger with the alcohol prep and used the lancet to create a controlled puncture. Up to 5 drops (~50 μL each) of free-flowing capillary blood were placed on the protein saver card. Participants were instructed to dry samples for 4‐12 hours and place the cards inside a study-provided biospecimen bag with a desiccant pack before shipping back to the study team. Samples were stored in −30°C freezers to ensure data quality before assay. Instructions for self-collecting DBS samples can be found in Tables S3 and S4 in Multimedia Appendix 1. mRNA assays were conducted on a subset of samples to detect and quantify gene expression in approximately 200 genes known to be involved in the regulation of inflammation. Whole-genome analyses were not conducted. The protocol for processing and analyzing DBS samples is described in 2 previous studies [36,37].

#### Microbiome Sample

Participants were asked to attach the OM-ACI accessory to their toilet, capture a fecal sample, and use the gloves and spatula to place a sample into the sample tube, which suspended the sample in stabilizing liquid (see Table S4 in Multimedia Appendix 1). Once samples were received, they were then stored at −80°C until DNA extraction and sequencing. Samples were then submitted for DNA extraction, library preparation, and sequencing. Specifically, DNA was extracted using the QIAGEN DNeasy PowerSoil Pro protocol (QIAGEN) [44], and DNA concentration was verified using the Qubit dsDNA HS Assay Kit [45,46]. Libraries were prepared according to the QIAGEN FX DNA Library Preparation Kit (QIAGEN) [46]. Quality and quantity of the finished libraries were assessed using an Agilent Tapestation [47] and Qubit dsDNA HS Assay Kit [45], respectively. For 16S rRNA gene amplicon sequencing, paired-end, 300 bp sequencing was performed using the Illumina NextSeq 2000 Sequencer [48] and a P1 600 cycle cartridge, or the Illumina MiSeq Sequencer and a MiSeq 600 bp (v3) sequencing cartridge [49]. For the WGS, paired-end 150-bp sequencing was performed using the Illumina NovaSeq X Plus [50].

To determine whether samples were successfully sequenced, we used a combination of criteria: (1) number of reads per sample and (2) Phred quality of reads. For the 16S rRNA amplicon sequencing, the cutoff was 10,000 reads per sample, and for WGS, the cutoff was 20 million. For the quality of reads, we averaged the per-read Phred quality score and used a cutoff of >30. A composite score of both criteria was then used to determine whether samples were successfully sequenced. Overall, 1741 samples were submitted for 16S rRNA amplicon sequencing, and 1589 samples were submitted for WGS. Figure S2 in Multimedia Appendix 1 illustrates quality of reads.

### Retention Efforts

To aid in participant retention, automated messages through REDCap reminded participants of upcoming, current, or overdue study activities. In addition, the study team was accessible by email and phone during normal business hours, prioritized responding to participants’ questions within 1‐2 business days, ensured timely milestone payments (ie, within 2 weeks of milestone completion), and mailed birthday cards to encourage engagement with the study (Figure S3 in Multimedia Appendix 1).

### Statistical Analysis

We calculated descriptive statistics to characterize the sample across demographic and clinical domains, and the study’s feasibility outcomes. Next, we conducted binary logistic regressions to evaluate whether age and depressive symptom severity at screening (PHQ-9 [27]) predicted completion of baseline activities. We then conducted a series of binary logistic regressions to evaluate predictors of data completion and data quality. Predictor variables included recruitment source (Craigslist, UW–Madison, and all other sources), demographic characteristics (ie, age, gender, race, ethnicity, income, and education), and depressive symptom severity (baseline Patient Health Questionnaire-8 [PHQ-8] [51]). Demographic variables were included as covariates in regression models when depressive symptoms were the predictor of interest.

Binary outcome variables (0=no, 1=yes) were created to indicate whether participants had completed questionnaire data, behavioral data (internet video, video log, and MST), and biological data (DBS and microbiome) at baseline, post treatment, or 17-week follow-up. Similarly, binary variables (0=no, 1=yes) were created to indicate whether questionnaire, behavioral (internet video, video log, and MST), and biological data (DBS and microbiome) were of sufficiently high quality to analyze as intended at week 4 or 17-week follow-up. Questionnaire data were determined to be of high quality if participants passed an attention check item (ie, not endorsing “I have been randomly selecting responses on this survey”). Video data were considered high-quality if data met quality cutoffs based on facial luminance, facial contrast, interocular head scale, voice activity detection, and transcription validity. High-quality MST data were defined as providing data for at least 75% of trials with scores within 3 SD of the mean. Biological data were considered high-quality if samples were of sufficient quality to be analyzed. For both completion and quality, participants were coded as 1 (ie, completed, high-quality) for a given type of data if any data of a given type (ie, questionnaire, behavioral, and biological) were completed or of high quality at either week 4 or at 17-week follow-ups, respectively. Results of regression analyses were reported in terms of odds ratios (ORs) with 95% CIs. Data from duplicate enrollees were excluded (n=5) from all analyses.

### Ethical Considerations

This study was approved by the Health Sciences Institutional Review Board (IRB) at the UW–Madison (approval number: 2021‐0991). The study was conducted in accordance with the Declaration of Helsinki and relevant institutional guidelines. All participants provided electronic informed consent prior to participation.

## Results

### Participant Recruitment

Participants enrolled in the BeWell Study were adults (n=1156, mean age 38.69, SD 12.14 years; see Table 1) recruited remotely across all 50 states (see Figure S4 in Multimedia Appendix 1). Sample demographics are reported in Table S7 in the Multimedia Appendix 1. The majority of participants self-reported as White (861/1123, 76.67%), non-Hispanic (1003/1124, 89.23%), and women (869/1151, 75.50%).

At screening, the sample had a mean PHQ-9 score of 10.67 (SD 4.90), indicating moderate depressive symptoms. Clinical interviews [32] determined that 69.55% (804/1156) of participants met criteria for a current depressive disorder and 84.78% (980/1156) met criteria for a past depressive disorder (eg, major depressive disorder and persistent depressive disorder). Of these, 43.86% (507/1156) met criteria for a current major depressive episode, and 77.94% (901/1156) met criteria for a past major depressive episode. The remainder of the sample met PHQ-9 screening criteria (ie, ≥5) but did not meet criteria for a *DSM-5* depressive disorder.

Participants were recruited from May 31, 2022, to December 11, 2023. Craigslist postings (18,169/22,782, 79.75%), ads on social media or online platforms (1011/22,782, 4.44%), and UW–Madison email advertisements (912/22,782, 4%) were the most effective forms of recruitment for participants who took the screener. Craigslist postings (694/1154, 60.14%), UW–Madison email advertisements (107/1154, 9.27%), and other recruitment methods (85/1154, 7.37%) were the most successful for the enrollment of eligible participants (see Figure S5 in Multimedia Appendix 1).

**Table 1.** Summary of data quality^a^.

Measure	Baseline	Week 1	Week 2	Week 3	Post treatment	17-Week
Questionnaire, n/N (%)	—^b^	1012/1025 (98.73)	943/956 (98.64)	928/941 (98.62)	986/1001 (98.50)	926/946 (97.89)
Behavioral, n/N (%)						
SEA^c^ task	975/1035 (94.20)	—	—	—	773/857 (90.20)	731/796 (91.83)
Video log	938/1035 (90.63)	780/880 (88.64)	709/807 (87.86)	690/801 (86.14)	725/857 (84.60)	678/796 (85.18)
MST^d^	1056/1121 (94.20)	—	—	—	871/924 (94.26)	818/868 (94.24)
Biological, n/N (%)						
Blood (at least one analysis)	857/859 (99.77)	—	—	—	—	849/859 (98.84)
Blood (all analyses)	816/859 (94.99)	—	—	—	—	807/859 (93.95)
Microbiome (16S)^e^	865/870 (99.43)	—	—	—	—	867/869 (99.77)
Microbiome (WGS)^f^	793/794 (99.87)	—	—	—	—	793/793 (100)

a This table displays the amount of high-quality data out of the total number of returned data at each time point, with corresponding percentages in parentheses.

b Data were not collected at that time point. Due to a technical error, some participants received the stimulus-elicited affect task during weeks 1‐3. Thus, their data are not displayed.

c SEA: stimulus-elicited affect.

d MST: Mnemonic Similarity Task.

e 16S: 16S rRNA amplicon sequencing.

f WGS: whole-genome sequencing.

### Participant Retention and Activity Completion

The screener was taken a total of 52,052 times. Of those, 1156 (2.22%) participants who started the screener were enrolled and randomized. Of the enrolled participants, 971 (84%) participants completed one or more measures at 17-week follow-up and were considered retained. A summary of activity completion is captured in Figure 3 (CONSORT [Consolidated Standards of Reporting Trials] diagram) and Figure S6 in Multimedia Appendix 1, with a summary of the experimental overview in Figure 1.

Completion of online questionnaires was ≥81.40% across the 6 time points of the study. Of the participants who completed questionnaires (which occurred prior to the video EMA being administered), ≥84.14% (796/946) of participants completed the SEA task and the video log task. Of the participants who completed questionnaires, ≥91.75% (868/946) of participants completed the MST across the 3 time points at which it was collected (baseline, week 4, and 17-week follow-up). Completion of biological samples was ≥99.05% (1145/1156) and ≥75.17% (869/1156) at the 2 time points (baseline and 17-week follow-up), respectively. No participants requested or scheduled a remote walk-through of data collection procedures from the study team. For a more detailed description of distribution of participant completion rates across data collection activities, see Figure S7 in the Multimedia Appendix 1. The US $50 bonus for those who completed or attempted to complete all measures but reported technical or shipping issues was awarded to 61.51% (711/1156) of participants.

**Figure 3.**
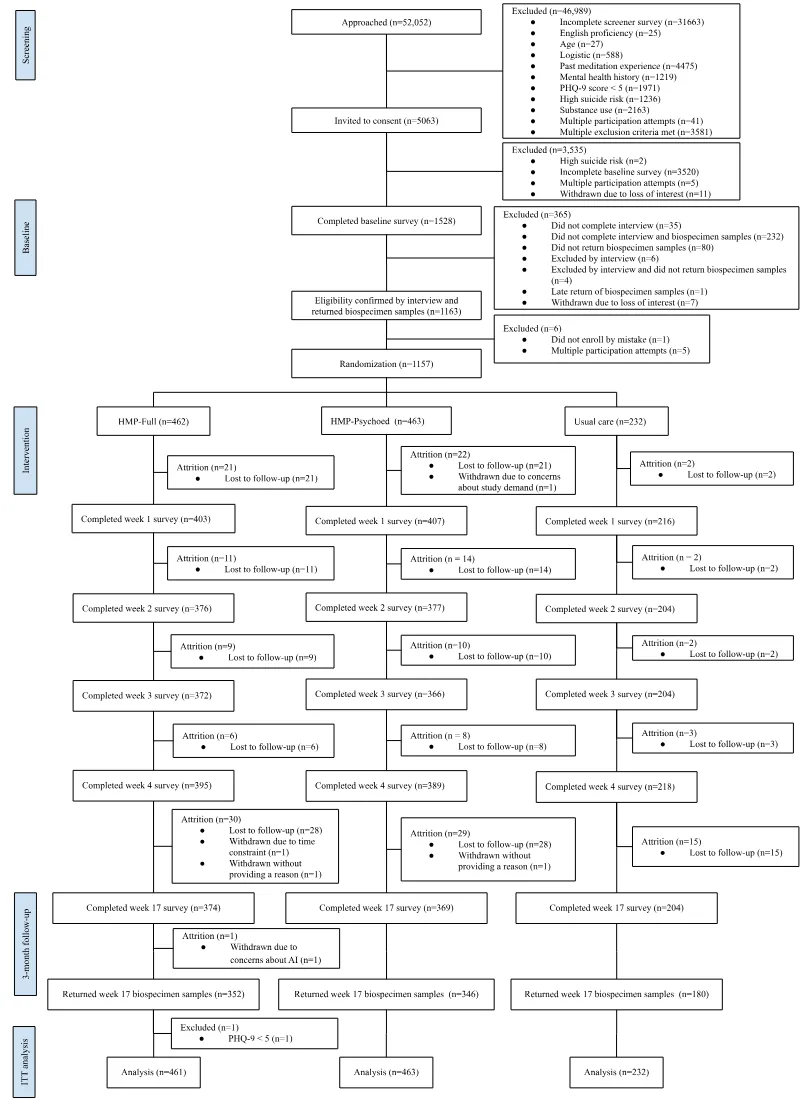
CONSORT (Consolidated Standards of Reporting Trials) diagram*.* This figure illustrates the flow of participants through each phase of the Behavior, Biology, and Well-being (BeWell) Study—a 3-arm, randomized, decentralized clinical trial. A total of 52,052 participants engaged with the online screener. Of these, 1156 participants consented and were randomized to 1 of 3 study conditions: HMP-Full: Healthy Minds Program app with full content (meditation and didactic material; n=461). HMP-Psychoed: Healthy Minds Program app with didactic material only (n=463). UC: participants were not assigned to any digital interventions but were freely available to continue in their usual care (n=232). All participants received a list of national mental health resources (eg, 9-8-8). Reasons for exclusion are described at each step. The diagram details the number of participants excluded at each stage of the 4-week intervention and the 17-week follow-up and the reasons for exclusion (eg, ineligibility, noncompletion of baseline tasks, and withdrawal). ITT; intention-to-treat; PHQ-9: Patient Health Questionnaire-9.

### Demographic and Clinical Characteristics at Screening Predicting Baseline Completion

Associations between age and depressive symptom severity at screening predicting completion of baseline measures are reported in Table S8 in Multimedia Appendix 1. Age was not a significant predictor of completion for any baseline measure (*P*=.39-.98). However, higher PHQ-9 scores at screening were consistently associated with lower odds of completing all baseline study measures after adjusting for screening age (questionnaire: OR 0.98, 95% CI 0.97‐0.99; *P*<.001; SCID interview: OR 0.97, 95% CI 0.96‐0.98; *P*<.001; DBS: OR 0.97, 95% CI 0.95-0.98; microbiome sample collection: OR 0.97, 95% CI 0.95‐0.98; *P*<.001; video log task: OR 0.96, 95% CI 0.95-0.98; *P*<.001; SEA task: 0.96, 95% CI 0.95-0.98; *P*<.001; and MST completion: OR 0.97, 95% CI 0.95‐0.98; *P*<.001). This suggests that eligible participants with greater depressive symptom severity at screening were less likely to complete baseline study procedures and be randomized.

### Data Quality

High-quality questionnaires were provided ≥97.89% (926/946) at all collected time points. At all time points, the percentage of collected data that was high-quality was ≥90.20% (773/857) for the SEA task and ≥84.60% (725/857) of video log data. Across all collected time points, ≥94.20% (1056/1121) MST data were considered high-quality. DBS samples were analyzable in ≥98.84% (849/859) of cases for at least one analyte and fully analyzable in ≥93.95% (807/859) of cases. 16S rRNA gene amplicon succeeded for ≥99.43% (865/870) of samples and microbiome WGS analyses succeeded in ≥99.87% (793/794) of samples. For a summary of data quality across time points and measures, see Table 1. More details of data quality can be found in the “Methods” section for each corresponding modality.

### Recruitment Source as a Predictor of Data Completion and Quality

Recruitment from Craigslist and the UW–Madison listserv, when compared to all other recruitment sources, was not predictive of the completion of week 4 or week 17 follow-ups, behavioral, or biological measures (*P*=.12-.67). Similarly, recruitment source did not predict questionnaire quality at week 4 or week 17 follow-ups (*P*=.31-.97). There was insufficient variability in week 4 or week 17 follow-ups behavioral and biological data quality (ie, almost all participants provided high-quality data for one or more measures) to examine predictors. For a summary of results, see Table 2. A more detailed overview of the association between recruitment source and completion and quality for each measure and time point can be found in Tables S9 and S10 in Multimedia Appendix 1.

**Table 2.** Recruitment source as predictors of week 4 or week 17 follow-up questionnaire, behavioral, and biological measure completion and quality^a^.

Predictors	Questionnaire			Behavioral		Biological	
	n	OR^b^ (95% CI)	*P* value	OR (95% CI)	*P* value	OR (95% CI)	*P* value
Completion							
Craigslist	1154	1.18 (0.80‐1.72)	.39	1.20 (0.85‐1.69)	.30	0.80 (0.60‐1.05)	.12
UW^c^–Madison	1154	1.16 (0.62‐2.43)	.67	1.35 (0.73‐2.72)	.37	1.18 (0.74‐1.95)	.50
Quality							
Craigslist	1032	1.86 (0.56‐6.48)	.31	—^d^	—	—	—
UW–Madison	1032	1.04 (0.20-19.15)	.97	—	—	—	—

a n=1154 for completion analyses (task completers with recruitment source data, 2 participants lost due to this question being added after the start of the study); n=1032 for quality analyses (task completers with high-quality data and recruitment source data). OR=odds ratios from logistic regressions predicting task completion or high-quality data by recruitment source. Craigslist and UW–Madison refer to participants recruited via those channels and are compared to all others (reference group). Odds ratios >1 reflect higher odds and <1 reflect lower odds. No effects were statistically significant (*P*<.05).

b OR: odds ratio.

c UW: University of Wisconsin.

d Insufficient variance to predict quality due to high levels of behavioral and biological data quality post intervention.

### Demographics as a Predictor of Completion and Quality

#### Overview

Several associations emerged between demographics (age, gender, race, ethnicity, income, and education) and data completion and quality (see Table S11 in Multimedia Appendix 1). A more detailed summary of the association between demographics and completion and quality for each measure and time point can be found in Tables S12 and S13 in Multimedia Appendix 1.

#### Age

The odds of completing study measures at week 4 or week 17 follow-ups increased with age for all categories of data collected (ie, questionnaire, behavioral, and biological). Higher age was associated with increased likelihood of completing postintervention questionnaires (OR 1.03, 95% CI 1.02-1.05; *P*<.001), behavioral measures (OR 1.02, 95% CI 1.01-1.04; *P*=.003), and biological measures (OR 1.03, 95% CI 1.01-1.04; *P*<.001). However, age was not significantly predictive of higher-quality responses to questionnaires (*P*=.08).

#### Gender

Participants who identified as a gender categorized as “other,” compared to the gender reference category of “woman,” demonstrated significantly lower odds of completing week 4 or week 17 follow-up questionnaires (OR 0.38, 95% CI 0.17‐0.92; *P*=.02). Categories of “man” and “other,” compared with the reference group of “woman,” were not predictive of completion or quality of questionnaires at week 4 or week 17 follow-ups (*P*=.07-1.00).

#### Race and Ethnicity

Race and ethnicity were not predictive of the completion of questionnaire, behavioral, or biological measures at week 4 or week 17 follow-ups (*P*=.25-.93). The racial and ethnic category of non-Hispanic White, compared to all other race and ethnic categories, was predictive of a greater likelihood of providing high-quality questionnaire data at week 4 or week 17 follow-ups (OR 8.86, 95% CI 2.21-59.09; *P*=.006).

#### Education

College graduates, compared to participants who reported not having graduated college, were significantly more likely to complete week 4 or week 17 follow-up questionnaires (OR 1.67, 95% CI 1.13-2.48; *P*=.01), behavioral tasks (OR 1.56, 95% CI 1.08-2.23; *P*=.02), and biological tasks (OR 1.58, 95% CI 1.18-2.11; *P*=.002). College graduates, compared to participants who reported not having graduated from college, were not more likely to provide high-quality questionnaire data at week 4 or week 17 follow-ups compared to those who had not graduated from college (*P*=.89).

#### Income

Household income greater than the 2022 census-reported national median of US $75,000 [52], compared to household income lower than the national median, was not predictive of the completion of questionnaire, behavioral, or biological data at week 4 or week 17 follow-ups (*P=*.68-.93). Similarly, household income was not predictive of questionnaire data quality (*P*=.99).

### Depression Severity as a Predictor of Data Completion and Quality

Baseline depression severity (ie, PHQ-8), controlling for all other demographic information, was not predictive of the completion of any category of study activity at week 4 or week 17 follow-ups (*P*=.16-.32). Similarly, depression severity was not associated with questionnaire data quality at week 4 or week 17 follow-ups (*P*=.90).

## Discussion

This study reports the methodology developed to incorporate a multimodal battery of psychological, behavioral, and biological measures within a large-scale (n=1156), 3-arm, randomized, longitudinal DCT testing a DMHI—the HMP app—in a national sample of adults selected for clinically elevated depressive symptoms. Secondarily, to inform future research in this area, we assessed the feasibility of this approach by examining data completion and quality across modalities, as well as recruitment sources, demographics, and depressive symptoms as predictors of these outcomes. While advancements have enabled mental health interventions to be delivered in an individual’s real-world context via DMHIs, and therefore incorporated into DCTs, methodological challenges remain for researchers aiming to rigorously test them in the field with objective behavioral and biological measures. The methodology developed for the BeWell Study provides one approach to addressing these methodological challenges. Results were largely encouraging; participants completed a multimodal battery of measures entirely remotely (≥68% completion rate across all modalities and time points), remotely collected data were of high quality (≥84% analyzable data across all modalities and time points), and high participant retention (971/1156, 84%) was maintained. To our knowledge, the BeWell Study is the largest DCT to use a fully remote, multimodal battery of both self-report and objective psychological, biological, and behavioral measures in a sample with clinically elevated depressive symptoms testing a DMHI. In addition to describing and contextualizing the methodology’s strengths, we consider its limitations and feasibility.

We successfully recruited a large sample of participants with elevated depressive symptoms, primarily using Craigslist, a US-based advertising website, and a university campus listserv. Both methods were low-cost and together accounted for 69.41% (801/1154) of enrolled participants. Craigslist, in particular, enabled us to recruit from specific regions of the United States, which facilitated intentional outreach to areas with large racial and ethnic minority populations, and the enrollment of participants from all 50 US states. Although we were ultimately unable to match the US Census with racial and ethnic proportions, 31.12% (346/1112) of participants did not identify as non-Hispanic White, which is an identical proportion reported in a recent systematic review of randomized trials of app interventions for depression and/or anxiety [53] and higher than the proportion reported in a systematic review of randomized trials of mindfulness-based interventions [54]. Notably, we did not find evidence that recruitment sources were associated with measure completion or quality. This finding can allay concerns that participants from recruitment sources that are not primarily designed for clinical trial recruitment (eg, Craigslist) may be less likely to remain engaged. Together, results suggest that recruitment from Craigslist and university campus listservs can yield large samples with minimal cost without sacrificing measure completion and quality.

An important question, but perhaps especially in DCTs, is the degree to which participant characteristics influence measure completion and data quality. We found older participants were more likely to complete measures across modalities (ie, questionnaire, behavioral, and biological), although age was not associated with data quality. College graduates were more likely to complete all measures. Individuals with a gender coded as “other” were less likely than the reference group of women-identified participants to complete the questionnaire data. However, this result warrants caution, as only 1.06% of the study sample identified as the gender category “other.” Differences did not emerge by gender for the other modalities or for data quality. Non-Hispanic White individuals were more likely to pass the attention check than other racial and ethnic categories but no more likely to complete measures. Depression severity at screener predicted lower odds of completing all baseline measures—an enrollment requirement. However, among enrolled participants, baseline depression severity did not predict measure completion or data quality.

The findings highlight that some disparity-linked identities (eg, noncollege graduates and other gender) were associated with attrition, whereas others (eg, race and ethnicity) were not. The effect sizes associated with data completion for other genders (OR 0.38, 95% CI 0.17-0.92, for questionnaires) and educational attainment (ORs 1.56, 95% CI 1.08-2.23, to OR 1.67, 95% CI 1.13-2.48, across measurement domains) are both small, based on conventional guidelines [55]. Nonetheless, they indicate room for improvement regarding engaging the full range of participants throughout the course of a DCT. Findings related to prediction of data quality partially replicate prior work, which has found that some racial and ethnic minority groups are more likely to have data flagged for quality reasons, although other associations (lower-quality data from younger participants) were not replicated [56]. Therefore, it will be important for future DCTs to oversample underrepresented groups to ensure that the sample is not further imbalanced if data are omitted due to quality concerns. Though greater depressive symptoms were associated with a lower likelihood of completing baseline measures and eligibility for enrollment, the lack of association between depression severity and both completion and data quality among participants enrolled in the study is encouraging. This suggests, among enrolled participants, study procedures were equally feasible across the range of symptom severity of enrolled participants, which is notable, given that anhedonia and low motivation are cardinal symptoms of depression. Nonetheless, as discussed below, results must be interpreted knowing that only participants who completed baseline measures were randomized. This created a selection bias among participants enrolled, with implications for the external validity of results.

The BeWell protocol had several strengths, most importantly, demonstrating the feasibility of gathering multimodal data from a national sample at scale, within a fully remote, DCT. Objective behavioral and biological measures, in particular, present substantial challenges for collection and processing within a fully remote design. Yet, such measures are crucial in improving the level of rigor at which DMHIs can be assessed. In particular, we note the novelty of including video EMA data and biological samples remotely, which provide important, objective insights into participants’ well-being sampled within their environment. The original development of the Teddy app took approximately 1 month to develop by a senior software engineer. That the Teddy app is open source and available for researchers to use and replicate within their own studies is a substantial contribution and additional strength [33]. Sharing the platform in this way reduces redundant redevelopment and minimizes waste of financial resources. We note that the use of reloadable debit cards, used to pay participants more quickly than with checks (ie, funds immediately available upon processing), our procedure for sending and receiving biological samples through FedEx, and clear participant instructions for biological sample collection were some features that may have contributed to measure completion. Nonetheless, there are several important limitations. First, completion of some measures, especially the video EMA task, proved somewhat challenging: participants were directed to it only after completing REDCap questionnaires. Some participants completed questionnaires on their computer, which required them to switch to their phone to complete video EMA tasks. Future studies should streamline this process (eg, integrating the video EMA into REDCap survey flow).

We were not able to recruit racial and ethnic minority participants in proportion to the US Census. Future studies may need to combine the low-cost recruitment methods (eg, Craigslist) with higher-cost alternatives that offer targeted recruitment and oversampling based on demographics. This could include the use of online advertising that is based on user demographics or the use of commercial recruitment services that guarantee recruitment based on demographic and other variables (eg, symptom severity) or partnering with community-based organizations. Third, we saw evidence that some baseline demographic characteristics were associated with both measure completion and data quality. This highlights the importance of future efforts aimed at ensuring high rates of completion across demographic groups. Future work may also examine methods to increase data quality. The length of the assessment battery in the BeWell Study (30-90 minutes) may have contributed to lower questionnaire data quality for some groups. Thus, future studies may benefit from a more streamlined set of measures to combat inattentive responding.

Additional important limitations are questions regarding the degree to which we can reasonably generalize from our sample of participants to the broader population of US adults with elevated depression symptoms. Generalizability could be limited due to our recruitment methods (eg, Craigslist and UW–Madison campus emails). These recruitment sources may not reflect the broader depressed population, and generalizability may also be constrained by regulatory, ethical, or logistical differences across countries that could limit replication of this protocol elsewhere. In addition, only a very small fraction of individuals who started the screener were ultimately randomized (ie, 1156/52,052, 2.22%). Furthermore, because depression severity significantly predicted a lower likelihood of completing baseline measures—a necessary step for enrollment—the screening-to-enrollment ratio in this study poses a threat to external validity. Even so, selecting participants who were able to complete baseline measures, which were repeated throughout the study, strengthened its internal validity, presumably by minimizing missing data post randomization. Potential bias and decreased generalizability related to the screening-to-enrollment ratio is a long-standing challenge in clinical trials [57] and certainly a crucial limitation of the current methodology and broader trial. Although our study had relatively few exclusion criteria (eg, did not exclude based on most forms of psychiatric comorbidity), participants were very likely excluded for a variety of unmeasured factors (eg, conscientiousness) linked to their ability to complete the steps required to be enrolled (eg, returning biological samples). At a minimum, retention rates were certainly increased, and fraud related to repeated enrollment minimized, by requiring all participants to complete a synchronous diagnostic interview session and to return biological samples prior to randomization. More clearly determining why participants are or are not willing to continue with enrollment in multimodal DCTs will be valuable to investigate in future studies. In addition, using methods explicitly designed to determine the representativeness of the enrolled sample a priori would also be valuable.

These limitations notwithstanding, this study demonstrates that a multimodal assessment approach is feasible within DCTs. Specifically, the study showed that digital intervention trials can include the kinds of behavioral and biological measures that have been historically limited to the laboratory setting. Although behavioral tasks have been used in fully remote digital health trials previously [58], our study demonstrated the feasibility of gathering a broad set of behavioral and biological measures within a sample with elevated symptoms of depression. Multimodal approaches such as this are essential for understanding the effects of digital interventions on key psychological, behavioral, and biological aspects of depression that cannot be measured using self-report or other measures typically used in DCTs. Multimodal assessment approaches have the potential to yield more effective and more personalized digital interventions to reduce the burden of disease associated with depression and other mental illnesses.

## Supplementary material

10.2196/92589 Multimedia Appendix 1 Data quality metrics, recruitment and enrollment characteristics, completion rates, study measures and procedures, and regression analyses of predictors of enrollment and data quality.

10.2196/92589 Checklist 1 CONSORT Checklist.

## Abbreviations

AUDIT: Alcohol Use Disorder Identification Test
BeWell: Behavior, Biology, and Well-being
CONSORT: Consolidated Standards of Reporting Trials
CRP: C-reactive protein
DBS: dried blood spot
DCT: decentralized clinical trial
DMHI: digital mental health intervention
*DSM-5*: *Diagnostic and Statistical Manual of Mental Disorders* (Fifth Edition)
DUDIT: Drug Use Disorders Identification Test
EMA: ecological momentary assessment
HIPAA: Health Insurance Portability and Accountability Act
HMP: Healthy Minds Program
HMP-Full: Healthy Minds Program app with full content
HMP-Psychoed: Healthy Minds Program app with didactic material only
IL: interleukin
IRB: Institutional Review Board
MST: Mnemonic Similarity Task
NAMI: National Alliance on Mental Illness
OR: odds ratio
PHQ-8: Patient Health Questionnaire-8
PHQ-9: Patient Health Questionnaire-9
SCID-5-RV: Structured Clinical Interview for *DSM-5* Research Version
SEA: stimulus-elicited affect
TNF-α: tumor necrosis factor α
UW: University of Wisconsin
VAD: voice activity detection
WGS: whole-genome sequencing
