# Supplementary material for: Incorporating Objective Behavioral and Biological Measures in a Large-Scale Decentralized Mobile Health Trial for Depression: Randomized Controlled Trial
Source: JMIR Ment Health. 2026 Oct 9;13:e92589. doi: 10.2196/92589 (PMC13698800; doi:10.2196/92589)
Supplement: Supplementary file 1 [file mental-v13-e92589-s001.docx]

**Figure S1**. Measures of Video EMA Quality

*Note*. This figure illustrates various visual properties of the Video Log and Stimulus Elicited Affect (SEA) visual data. On the top, we show the interocular head scale (where 1 indicates that the distance from eye to eye is 15 % of the frame width), facial luminance (on an grayscale from 0 to 250), facial contrast (based on Michelson Contrast equation, from 0 to 1), tracker confidence (based on the Affdex 2.0 algorithm’s confidence, ranging from 0 to 100%), pitch (positive values indicate face pointing up relative to camera), yaw (rotation around the neck ), and roll (rotation around the nose), and attention (ranging from 0 to 100%). The range for radian values is from -2π to 2π.

**Figure S2.** Microbiome Sample Sequence Quality

*Note*. Overview of microbiome sample sequence quality for 16S rRNA (A and B) and shotgun whole genome sequencing (WGS) (C and D). (A): Samples ranked by total number of reads the dotted line indicates the 10,000 read cutoff used as a measure of success for 16S rRNA amplicon sequencing. (B): Distribution of the average Phread score per read for 16S rRNA amplicon sequencing. (C): Samples ranked by total number of reads the dotted line indicates the 20-million read cutoff used as a measure of success for WGS. (D): Distribution of the average Phred score per read for WGS.

**Figure S3.** Birthday Cards for Participants

*Note*. This figure illustrates the birthday cards sent to participants who participated in the Behavior, Biology, and Wellbeing (BeWell) Study.

**Figure S4.**Participant Enrollment by State

*Note.* This figure is a heat map of participant recruitment density across the 50 U.S. states. The figure displays the number of participants screened in each state, with colors closer to red indicating greater participant density

Figure S5. Recruitment Source Breakdown

*Note.* This figure illustrates the sources through which screened and enrolled participants learned about the study, as self-reported by participants. *Ads on Broadcast media* = local ad, newspaper ad, television ad, podcast appearances, *E-mail =* UW – Madison email listserv recruitment, *Research Participant Recruitment Platforms =* ResearchMatch, Prolific, Power, *Website of Mental Health Organizations =* National Alliance on Mental Illness (NAMI) and Mental Health America, *Flyer, Letter, or Brochure =* Flyers, letters and brochures sent to local and national mental health clinics, flyers posted locally in Madison, WI, *Social Media or Online Forums* = Facebook, Twitter, Instagram, Reddit *Craigslist =* Nationally-posted Craigslist advertisements, *Clinicaltrials.gov* = advertisement on clinicaltrials.gov website, *Other =* any source other than those provided in the survey*, Multiple channels* = recruitment from more than one source listed above

**Figure S6***.* Participant Task Completion Across Study Timepoints (n = 1,156)

*Note.* Bar plot shows the number of participants completing each task: self-report survey, Video Log, stimulus-elicited affect (SEA), Mnemonic Similarity Task (MST), dried blood spot (DBS) sample, and microbiome sample at Baseline, Intervention (Week 1-4), and 17-week follow-up. SEA and MST were not assessed during Weeks 1-3, and DBS and microbiome were only collected at Baseline and 17-week follow-up. Counts reflect observed completion, with missing data treated as non-completion. Zero‑completion timepoints are unlabeled for clarity.

**Supplementary Figure S7.** Distribution of Participant Completion Rates Across Data Collection Measures

*Note***.** Percentages indicate the proportion of enrolled participants (N = 1,156) completing available data collection activities across baseline, Weeks 1–4, and 17-week follow-up. Surveys were administered at all time points; biospecimens (dried blood spot and microbiome samples) were collected at baseline and 17-week follow-up; and behavioral measures included video logs at all time points and stimulus-elicited affect and mnemonic similarity tasks at baseline, Week 4, and 17-week follow-up. Stacked bars show the proportion of participants completing each number of measures within each data collection category.

**Table S1.** Participant Compensation Schedule

| **Study activity** | **Time point** | **Compensation, $** |
| --- | --- | --- |
| Dried blood spot sample | Baseline | 20 |
| Stool sample | Baseline | 30 |
| Questionnaires and behavioral tasks^a^ | Weeks 1-3 | 10 per week |
| Questionnaires and behavioral tasks | Post-treatment (week 4) | 40 |
| Questionnaires and behavioral tasks | 17-week follow-up | 45 |
| Dried blood spot | 17-week follow-up | 20 |
| Stool sample | 17-week follow-up | 30 |
| Completion of all study activities - Bonus | 17-week follow-up | 50 |
| **Maximum total compensation** |  | **265** |

^a^ Participants could receive $10 per week during weeks 1 through 3, for a maximum of $30.

*Note:* Participants could receive up to $265 for completing all study activities. Payments were loaded onto participants’ Focus Blue cards at 3 points during the study (baseline, post-treatment, and 17-week follow-up) or were issued by check.

**Table S2**: List of Measures by Timepoint

| **Measure** | | **Screening** | **Baseline** | **Intervention (Week 1-3)** | **Post-treatment** | **17-week follow-up** |
| --- | --- | --- | --- | --- | --- | --- |
| **Psychological Symptoms** | |  |  |  |  |  |
|  | Alcohol Use Disorders Identification Test (AUDIT) [1] | X |  |  |  | X |
|  | Drug Use Disorders Identification Test (DUDIT) [2] | X |  |  |  |  |
|  | Generalized Anxiety Disorder Scale (GAD-7) [3] |  | X | X | X | X |
|  | Patient Health Questionnaire Depression Scale (PHQ-8) [4] |  | X | X | X | X |
|  | Patient Health Questionnaire Depression Scale (PHQ-9) [5] | X |  |  |  |  |
|  | Perceived Stress Scale (PSS-10) [6] |  | X |  | X | X |
| **PROMIS** | |  |  |  |  |  |
|  | PROMIS Sleep Disturbances – Computer Adaptive Version [7] |  | X | X | X | X |
|  | PROMIS Pain Interference Measure [8] |  | X |  | X | X |
|  | PROMIS Pain Intensity Measure [9] |  | X |  | X | X |
| **Physical Health** | |  |  |  |  |  |
|  | Environmental Toxin Questionnaire |  | X |  |  |  |
|  | Asthma Control Questionnaire (ACQ) [10] |  | X |  | X | X |
|  | Medication Questionnaires (health history, current health and medication use, and biospecimen collection questions) |  | X |  | X | X |
| **Well-Being and Other Psychological Measures** | |  |  |  |  |  |
|  | Climate Change Anxiety Scale (CCAS) [11] |  | X |  | X | X |
|  | Decentering Subscale of the Experiences Questionnaire [12] |  | X | X | X | X |
|  | Five Facet Mindfulness Questionnaire (FFMQ), Awareness Subscale [13] |  | X | X | X | X |
|  | Flourishing Measure [14] |  | X | X | X | X |
|  | Growth Mindset Scale for Well-Being [15] |  | X |  | X | X |
|  | Healthy Minds Index [16] |  | X |  | X | X |
|  | Inclusion of Nature in Self [17] |  | X |  | X | X |
|  | Life Orientation Test – Revised (LOT-R) [18] |  | X |  | X | X |
|  | NIH Toolbox Loneliness [19] |  | X | X | X | X |
|  | NIH Toolbox Meaning and Purpose [20] |  | X | X | X | X |
|  | Problematic and Risky Internet Use Screening Scale – 3 (PRIUSS-3) [21] |  | X |  | X | X |
|  | Perseverative Thinking Questionnaire (PTQ) [22] |  | X |  | X | X |
|  | Satisfaction with Life Scale (SWLS) [23] |  | X |  | X | X |
|  | Subjective Happiness Scale [24] |  | X |  | X | X |
| **Baseline-Only and Process Measures** | |  |  |  |  |  |
|  | Behavioral Risk Factor Surveillance System (BRFSS) – Adverse Childhood Experiences (ACE) Module [25] |  | X |  |  |  |
|  | Big Five Inventory - Conscientiousness Items [26] |  | X |  |  |  |
|  | Determinants of Meditation Practice Inventory – Revised [27] |  | X |  |  |  |
|  | Digital Working Alliance Inventory (DWAI) [28] |  |  | X | X | X |
|  | Lasting Effects Questionnaire |  |  |  |  | X |
|  | Marlowe-Crowne Social Desirability Scale (MCSD) [29] |  | X |  |  |  |
|  | Meditation and App Usage Questionnaire |  | X |  | X |  |
|  | Mindfulness Adherence Questionnaire |  |  | X | X | X |
|  | Mindfulness-Based Program Meditation-Related Adverse Effects Scale (MBP-MRAES) [30] |  |  |  | X |  |
|  | Pre and Post Practice Questions |  |  | X | X |  |
|  | Treatment Expectancies |  |  |  | X |  |
| **Clinical Interview** | |  |  |  |  |  |
|  | Structured Clinical Interview for DSM-5 (SCID-5), Depression Module [31] |  | X |  |  |  |
| **Behavioral Measures** | |  |  |  |  |  |
|  | Mnemonic Similarity Task [32] |  | X |  | X | X |
|  | Video Log Task |  | X | X | X | X |
|  | Stimulus Elicited Affect Task |  | X |  | X | X |
| **Biological Measures** | |  |  |  |  |  |
|  | Dried Blood Spot Sample [33] |  | X |  |  | X |
|  | Microbiome |  | X |  |  | X |

*Note:* “X” indicates administration at that time point.

**Table S3.** Participant Instructions for Dried Blood Spot Cards

**Table S4.** Participant Video Instructions for Dried Blood Spot and Microbiome Sample Collection

*Note.* Biosample collection kits contained QR codes which provided them with video instructions for collecting dried blood spot and microbiome samples in alignment with the Behavior, Biology, and Wellbeing (BeWell) Study protocol.

**Table S5.** Participant Instructions for Shipping Biological Samples

**Table S6.** Visual Quality Metrics in Detail

| **Video Type** | **Contrast** | | **Pitch (Rad)** | | **Yaw (Rad)** | | **Roll (Rad)** | |
| --- | --- | --- | --- | --- | --- | --- | --- | --- |
|  | Mean | SD | Mean | SD | Mean | SD | Mean | SD |
| **Internet Video** | 0.925 | 0.092 | 0.100 | 0.175 | 0.004 | 0.084 | 0.028 | 0.080 |
| **Video Log** | 0.930 | 0.086 | 0.178 | 0.168 | 0.015 | 0.108 | 0.031 | 0.087 |
|  | **Head Scale Interocular** | | **Luminance** | | **Tracker Confidence** | | **Attention** | |
|  | Mean | SD | Mean | SD | Mean | SD | Mean | SD |
| **Internet Video** | 1.867 | 0.420 | 112.400 | 23.446 | 96.211 | 1.504 | 96.296 | 5.832 |
| **Video Log** | 1.879 | 0.446 | 113.855 | 22.826 | 95.972 | 1.543 | 92.816 | 9.040 |

*Note.* This table presents the mean values for each visual quality metric, as well as the standard deviation (SD) of these metrics. The interocular head scale indicates the normalized distance between the eyes, where a value of 1 means the eye-to-eye distance is 15% of the frame width. Facial luminance captures the average brightness of the detected face on a grayscale from 0 (black) to 255 (white), while facial contrast—computed using the Michelson Contrast formula—quantifies the visual clarity of facial features on a scale from 0 to 1. Tracker confidence, based on the Affdex 2.0 algorithm, reflects the reliability of facial detection, ranging from 0% to 100%. The figure also shows head orientation in terms of pitch (up-down tilt), yaw (left-right turn), and roll (side tilt), with angular values spanning −2𝜋 to 2𝜋 radians. Lastly, attention estimates whether the subject is oriented toward the camera, ranging from 0 to 100%.

**Table S7.** Demographic and Clinical Characteristics of Study Population by Group

| **Characteristic** | | ***Non-Missing n*** | **Overall** | **HMP-Full** | **HMP-Psychoed** | **Usual Care** |
| --- | --- | --- | --- | --- | --- | --- |
|  |  |  | *n = 1,156* | *n = 461* | *n = 463* | *n = 232* |
| **Age in years, mean (SD)** | | 1,156 | 38.69 (12.14) | 38.35 (11.85) | 39.02 (12.18) | 38.72 (12.64) |
| **Gender, n/N (%)** | | 1,151 |  |  |  |  |
|  | Woman |  | 869 / 1,151 (75.50%) | 354 / 458 (77.29%) | 342 / 462 (74.03%) | 173 / 231 (74.89%) |
|  | Man |  | 245 / 1,151 (21.29%) | 93 / 458 (20.31%) | 103 / 462 (22.29%) | 49 / 231 (21.21%) |
|  | Other |  | 37 / 1,151 (3.21%) | 11 / 458 (2.40%) | 17 / 462 (3.68%) | 2 / 231 (3.90%) |
|  | Missing |  | 5 | 3 | 1 | 1 |
| **Race, n/N (%)** | | 1,123 |  |  |  |  |
|  | White |  | 861 / 1,123 (76.67%) | 337 / 448 (75.22%) | 351 / 455 (77.31%) | 173 / 221 (78.28%) |
|  | Black or African American |  | 104 / 1,123 (9.26%) | 37 / 448 (8.26%) | 46 / 455 (10.13%) | 21 / 221 (9.50%) |
|  | American Indian or Alaska Native |  | 8 / 1,123 (0.71%) | 2 / 448 (0.45%) | 5 / 455 (1.10%) | 1 / 221 (0.45%) |
|  | Asian or Asian American |  | 66 / 1,123 (5.88%) | 28 / 448 (6.25%) | 25 / 455 (5.51%) | 13 / 221 (5.88%) |
|  | Native Hawaiian or Other Pacific Islander |  | 2 / 1,123 (0.18%) | 0 / 448 (0.00%) | 1 / 455 (0.22%) | 1 / 221 (0.45%) |
|  | Multi-racial |  | 82 / 1,123 (7.30%) | 44 / 448 (9.82%) | 26 / 455 (5.73%) | 12 / 221 (5.43%) |
|  | Missing |  | 33 | 13 | 9 | 11 |
| **Non-Hispanic White, n/N (%)** | |  | 766 / 1,112 (68.88%) | 302 / 444 (68.02%) | 317 / 451 (70.29%) | 147 / 217 (67.74%) |
| **Ethnicity - Hispanic or Latino, n/N (%)** | | 1,124 |  |  |  |  |
|  | No |  | 1,003 / 1,124 (89.23%) | 399 / 451 (88.47%) | 406 / 449 (90.42%) | 198 / 224 (88.39%) |
|  | Yes |  | 112 / 1,124 (9.96%) | 48 / 451 (10.64%) | 40 / 449 (8.91%) | 24 / 224 (10.71%) |
|  | Prefer not to answer |  | 9 / 1,124 (0.80%) | 4 / 451 (0.89%) | 3 / 449 (0.67%) | 2 / 224 (0.89%) |
|  | Missing |  | 32 | 10 | 14 | 8 |
| **Education, n/N (%)** | | 1,147 |  |  |  |  |
|  | Graduated college |  | 674 / 1,147 (58.76%) | 275 / 458 (60.04%) | 271 / 459 (59.04%) | 128 / 230 (55.65%) |
| **Total family income, n/N (%)** | | 1,151 |  |  |  |  |
|  | Below median |  | 765 / 1,151 (66.46%) | 309 / 460 (67.17%) | 299 / 460 (65.00%) | 157 / 231 (67.97%) |
|  | Above median |  | 386 / 1,151 (33.54%) | 151 / 460 (32.83%) | 161 / 460 (35.00%) | 74 / 231 (32.03%) |
|  | Missing |  | 5 | 1 | 3 | 1 |
| **Baseline Clinical Characteristics** | |  |  |  |  |  |
|  | Screen PHQ-9 total, mean (SD) | 1,156 | 10.67 (4.90) | 10.62 (4.82) | 10.67 (4.92) | 10.76 (5.01) |
|  | Screen PHQ-9 total, median | 1,156 | 9.00 | 10.00 | 9.00 | 9.00 |
|  | Baseline PHQ-8 total, mean (SD) | 1,156 | 9.92 (4.76) | 10.07 (4.85) | 9.85 (4.68) | 9.79 (4.75) |
|  | Baseline PHQ-8 total, median | 1,156 | 9.00 | 10.00 | 9.00 | 9.00 |
|  | Baseline GAD-7 total, mean (SD) | 1,156 | 8.73 (4.96) | 8.96 (4.94) | 8.67 (4.97) | 8.37 (4.97) |
|  | Baseline GAD-7 total, median | 1,156 | 8.00 | 8.00 | 8.00 | 7.00 |
| **Structured Clinical Interview for DSM-5, n/N (%)** | |  |  |  |  |  |
|  | With Past Major Depressive Episode | 1,156 | 901 / 1,156 (77.94%) | 372 / 461 (80.69%) | 348 / 463 (75.16%) | 181 / 232 (78.02%) |
|  | With Current Major Depressive Episode | 1,156 | 507 / 1,156 (43.86%) | 207 / 461 (44.90%) | 198 / 463 (42.76%) | 102 / 232 (43.97%) |
|  | With Past Depressive Disorders | 1,156 | 980 / 1,156 (84.78%) | 397 / 461 (86.12%) | 382 / 463 (82.51%) | 201 / 232 (86.64%) |
|  | With Current Depressive Disorders | 1,156 | 804 / 1,156 (69.55%) | 324 / 461 (70.28%) | 313 / 463 (67.60%) | 167 / 232 (71.98%) |

*Note.* Demographic and clinical characteristics are reported for the full sample and by study condition. Values are presented as mean (SD) or n (%). PHQ-8 = Patient Health Questionnaire–8; GAD-7 = Generalized Anxiety Disorder–7; DSM-V = Diagnostic and Statistical Manual of Mental Disorders Fifth Edition.

**Supplementary Table S8.** A Summary of Logistic Regression Results: Age and PHQ-9 Score at screening as Predictors of Questionnaire, Video Tasks, Mnemonic Similarity Task, and Biological Sample Completion at Baseline

|  |  | **Questionnaire** | | | **SCID interview** | | | **Dried Blood Spot Card** | | | **Microbiome** | | | **Video Log Task** | | | **Stimulus Elicited Affect Task** | | | **Mnemonic Similarity Task** | | |
| --- | --- | --- | --- | --- | --- | --- | --- | --- | --- | --- | --- | --- | --- | --- | --- | --- | --- | --- | --- | --- | --- | --- |
| *Outcomes* | | *Odds Ratios^b^* | *CI^c^* | *P^d^* | *Odds Ratios* | *CI* | *P* | *Odds Ratios* | *CI* | *P* | *Odds Ratios* | *CI* | *P* | *Odds Ratios* | *CI* | *P* | *Odds Ratios* | *CI* | *P* | *Odds Ratios* | *CI* | *P* |
| **Model 1**^f^ | |  |  |  |  |  |  |  |  |  |  |  |  |  |  |  |  |  |  |  |  |  |
|  | Age | 1.00 | 0.99 – 1.00 | .54 | 1.00 | 0.99 – 1.00 | .54 | 1.00 | 1.00 – 1.01 | .42 | 1.00 | 1.00 – 1.01 | .66 | 1.00 | 0.99 – 1.00 | .37 | 1.00 | 0.99 – 1.00 | .37 | 1.00 | 0.99 – 1.01 | .98 |
| **Model 2**^g^ | |  |  |  |  |  |  |  |  |  |  |  |  |  |  |  |  |  |  |  |  |  |
|  | PHQ-9 at screening | 0.98 | 0.97 – 0.98 | **<.001** | 0.97 | 0.96 – 0.98 | **<.001** | 0.97 | 0.96 – 0.98 | **<.001** | 0.97 | 0.96 – 0.98 | **<.001** | 0.97 | 0.96 – 0.98 | **<.001** | 0.97 | 0.96 – 0.98 | **<.001** | 0.96 | 0.96 – 0.99 | **<.001** |

^a^Results from logistic regression models examining associates of age and PHQ-9 score at screening with completion of study tasks and biological sample collection among eligible participants invited to consent, excluding individuals removed due to multiple participations (N=5,090) at baseline. Completion of each task was coded as a binary outcome (1 = completed, 0 = not completed).
^b^OR = odds ratio.
^c^CI = 95% confidence interval.
^d^*p* values in bold indicate statistical significance (*p* < .05).
^f^Model 1 includes age.
^g^Model 2 adds depression (PHQ-9) at screening, controlling for age as covariates.

**Table S9.** A Summary of Logistic Regression Results: Recruitment Source as Predictors of Completion for Questionnaire, Video Tasks and Mnemonic Similarity Task Measures.

|  |  |  | **Baseline** | | | **Week 1** | | | **Week 2** | | | **Week 3** | | | **Post-treatment (Week 4)** | | | **17-week follow-up** | | |
| --- | --- | --- | --- | --- | --- | --- | --- | --- | --- | --- | --- | --- | --- | --- | --- | --- | --- | --- | --- | --- |
|  |  | *n* | *Odds Ratios* | *CI* | *P* | *Odds Ratios* | *CI* | *P* | *Odds Ratios* | *CI* | *P* | *Odds Ratios* | *CI* | *P* | *Odds Ratios* | *CI* | *P* | *Odds Ratios* | *CI* | *P* |
| **Questionnaire** | |  |  |  |  |  |  |  |  |  |  |  |  |  |  |  |  |  |  |  |
|  | Craigslist | 1,154 | – | – | – | 0.61 | 0.40 – 0.89 | **.01** | 1.10 | 0.80 – 1.49 | .56 | 0.90 | 0.66 – 1.22 | .51 | 1.02 | 0.72 – 1.44 | .91 | 0.88 | 0.64 – 1.19 | .41 |
|  | UW-Madison Email | 1,154 | – | – | – | 1.65 | 0.83 – 3.76 | .19 | 0.96 | 0.58 – 1.66 | .88 | 1.23 | 0.73 – 2.17 | .46 | 1.03 | 0.59 – 1.92 | .93 | 1.39 | 0.81 – 2.55 | .25 |
| **Stimulus Elicited Affect Task** | |  |  |  |  |  |  |  |  |  |  |  |  |  |  |  |  |  |  |  |
|  | Craigslist | 1,154 | 0.88 | 0.59 – 1.30 | .53 | – | – | – | – | – | – | – | – | – | 1.10 | 0.84 – 1.44 | .47 | 1.20 | 0.93 – 1.55 | .16 |
|  | UW-Madison Email | 1,154 | 1.50 | 0.75 – 3.42 | .29 | – | – | – | – | – | – | – | – | – | 0.93 | 0.60 – 1.48 | .75 | 1.63 | 1.03 – 2.66 | **.04** |
| **Video Log Task** | |  |  |  |  |  |  |  |  |  |  |  |  |  |  |  |  |  |  |  |
|  | Craigslist | 1,154 | 0.88 | 0.59 – 1.30 | .53 | 0.82 | 0.62 – 1.08 | .16 | 0.97 | 0.75 – 1.25 | .82 | 0.93 | 0.72 – 1.20 | .56 | 1.10 | 0.84 – 1.44 | .47 | 1.20 | 0.93 – 1.55 | .16 |
|  | UW-Madison Email | 1,154 | 1.50 | 0.75 – 3.42 | .29 | 2.22 | 1.28 – 4.12 | **.01** | 1.12 | 0.73 – 1.77 | .62 | 1.16 | 0.75 – 1.82 | .52 | 0.93 | 0.60 – 1.48 | .75 | 1.63 | 1.03 – 2.66 | **.04** |
| **Mnemonic Similarity Task** | |  |  |  |  |  |  |  |  |  |  |  |  |  |  |  |  |  |  |  |
|  | Craigslist | 1,154 | 0.89 | 0.43 – 1.76 | .74 | – | – | – | – | – | – | – | – | – | 0.95 | 0.71 – 1.28 | .76 | 0.82 | 0.62 – 1.07 | .15 |
|  | UW-Madison Email | 1,154 | 1.71 | 0.51 – 10.63 | .47 | – | – | – | – | – | – | – | – | – | 1.36 | 0.81 – 2.41 | .26 | 1.99 | 1.18 – 3.56 | **.01** |
| **Dried Blood Spot Card** | |  |  |  |  |  |  |  |  |  |  |  |  |  |  |  |  |  |  |  |
|  | Craigslist | 1,154 | 0.86 | 0.22 – 2.87 | .81 | – | – | – | – | – | – | – | – | – | – | – | – | 0.82 | 0.62 – 1.08 | .17 |
|  | UW-Madison Email | 1,154 | 1.02 | 0.19 – 18.85 | .98 | – | – | – | – | – | – | – | – | – | – | – | – | 1.23 | 0.77 – 2.03 | .41 |
| **Microbiome Samples** | |  |  |  |  |  |  |  |  |  |  |  |  |  |  |  |  |  |  |  |
|  | Craigslist | 1,154 | 1.127 × 10⁸ | <0.01 – NA | 1.00 | – | – | – | – | – | – | – | – | – | – | – | – | 0.80 | 0.61 – 1.06 | .12 |
|  | UW-Madison Email | 1,154 | 0.20 | 0.02 – 4.39 | .19 | – | – | – | – | – | – | – | – | – | – | – | – | 1.14 | 0.72 – 1.87 | .58 |

*Note*. Results from logistic regression models predicting completion of tasks and biological sample collection based on recruitment source. “Craigslist” refers to participants recruited from Craigslist. “UW-Madison” refers to participants recruited through the UW-Madison research study recruitment listserv. Odds ratios (OR), 95% confidence intervals (CI), and *p* values are reported. Quality was coded as a binary outcome (1 = good quality, 0 = poor quality), as described in the methods section. Missing values ("–") indicate that logistic regression was not conducted for that time point. *p* values in bold indicate statistical significance (*p* < .05). The not estimable confidence interval for recruitment source predicting microbiome WGS quality was related to zero cell counts

**Table S10.** Recruitment Source as Predictors of Data Quality

|  |  |  | **Baseline** | | | **Week 1** | | | **Week 2** | | | **Week 3** | | | **Post-treatment (week4)** | | | **17-week follow-up** | | |
| --- | --- | --- | --- | --- | --- | --- | --- | --- | --- | --- | --- | --- | --- | --- | --- | --- | --- | --- | --- | --- |
|  |  | *n* | *Odds Ratios* | *CI* | *P* | *Odds Ratios* | *CI* | *P* | *Odds Ratios* | *CI* | *P* | *Odds Ratios* | *CI* | *P* | *Odds Ratios* | *CI* | *P* | *Odds Ratios* | *CI* | *P* |
| **Questionnaire** | |  |  |  |  |  |  |  |  |  |  |  |  |  |  |  |  |  |  |  |
|  | Craigslist | 1,123 | – | – | – | 0.89 | 0.27 – 2.69 | .84 | 2.48 | 0.82 – 8.28 | .11 | 1.74 | 0.57 – 5.45 | .32 | 0.75 | 0.23 – 2.14 | .61 | 1.21 | 0.48 – 2.95 | .67 |
|  | UW-Madison Email | 1,123 | – | – | – | 1.29 | 0.25 – 23.62 | .81 | 0.33 | 0.10 – 1.50 | .10 | 0.58 | 0.15 – 3.77 | .48 | 5.29 x 10^6^ | 0.00 – NA | .99 | 2.07 | 0.75 – 37.46 | .48 |
| **Stimulus Elicited Affect Task** | |  |  |  |  |  |  |  |  |  |  |  |  |  |  |  |  |  |  |  |
|  | Craigslist | 1,034 | 0.92 | 0.53 – 1.56 | .76 | – | – | – | – | – | – | – | – | – | 0.52 | 0.30 – 0.84 | **.01** | 0.69 | 0.39 – 1.17 | .18 |
|  | UW-Madison Email | 1,034 | 1.51 | 0.60 – 5.07 | .43 | – | – | – | – | – | – | – | – | – | 1.65 | 0.71 – 4.80 | .30 | 0.96 | 0.45 – 2.38 | .93 |
| **Video Log Task** | |  |  |  |  |  |  |  |  |  |  |  |  |  |  |  |  |  |  |  |
|  | Craigslist | 1,034 | 0.61 | 0.38 – 0.94 | **.03** | 0.95 | 0.62 – 1.45 | .82 | 1.08 | 0.70 – 1.66 | .72 | 0.92 | 0.61 – 1.38 | .68 | 0.74 | 0.50 – 1.08 | .13 | 0.59 | 0.38 – 0.90 | **.02** |
|  | UW-Madison Email | 1,034 | 2.05 | 0.90 – 5.93 | .13 | 1.07 | 0.56 – 2.27 | .84 | 1.21 | 0.60 – 2.81 | .62 | 1.24 | 0.63 – 2.74 | .56 | 2.32 | 1.07 – 6.07 | .05 | 1.73 | 0.86 – 3.98 | .16 |
| **Mnemonic Similarity Task** | |  |  |  |  |  |  |  |  |  |  |  |  |  |  |  |  |  |  |  |
|  | Craigslist | 1,119 | 0.81 | 0.48 – 1.36 | .44 | – | – | – | – | – | – | – | – | – | 0.98 | 0.55 – 1.72 | .94 | 0.66 | 0.35 – 1.20 | .18 |
|  | UW-Madison Email | 1,119 | 3.41 | 1.05 – 20.98 | .09 | – | – | – | – | – | – | – | – | – | 1.04 | 0.44 – 3.06 | .94 | 1.37 | 0.54 – 4.63 | .56 |
| **Blood Analysis – At Least One Analyses** | |  |  |  |  |  |  |  |  |  |  |  |  |  |  |  |  |  |  |  |
|  | Craigslist | 858 | – | – | – | – | – | – | – | – | – | – | - | – | – | – | – | 0.16 | 0.01 – 0.84 | .08 |
|  | UW-Madison Email | 858 | – | – | – | – | – | – | – | – | – | – | – | – | – | – | – | 4.10 x 10^6^ | 0.00 – NA | .99 |
| **Blood Analysis – All Analyses** | |  |  |  |  |  |  |  |  |  |  |  |  |  |  |  |  |  |  |  |
|  | Craigslist | 858 | 0.54 | 0.26 – 1.04 | .07 | – | – | – | – | – | – | – | - | – | – | – | – | 0.32 | 0.15 – 0.62 | **.002** |
|  | UW-Madison Email | 858 | 4.63 | 0.99 – 82.72 | .13 | – | – | – | – | – | – | – | – | – | – | – | – | 5.70 | 1.22 – 101.49 | .09 |
| **Microbiome – 16S** | |  |  |  |  |  |  |  |  |  |  |  |  |  |  |  |  |  |  |  |
|  | Craigslist | 869 | 0.36 | 0.02 – 2.45 | .36 | – | – | – | – | – | – | – | - | – | – | – | – | 0.00 | NA – 1.37 x 10^183^ | 1.00 |
|  | UW-Madison Email | 869 | 5.46 x 10^6^ | 0.00 – NA | .99 | – | – | – | – | – | – | – | – | – | – | – | – | 5.93 x 10^6^ | 0.00 – NA | 1.00 |
| **Microbiome – WGS** | |  |  |  |  |  |  |  |  |  |  |  |  |  |  |  |  |  |  |  |
|  | Craigslist | 793 | 0.00 | NA – Inf | 1.00 | – | – | – | – | – | – | – | - | – | – | – | – | – | – | – |
|  | UW-Madison Email | 793 | 3.24 x 10^6^ | 0.00 – NA | 1.00 | – | – | – | – | – | – | – | – | – | – | – | – | – | – | – |

*Note***.** Results from logistic regression models predicting data quality of questionnaires, video tasks, and the Mnemonic Similarity Task based on recruitment source. Odds ratios (OR), 95% confidence intervals (CI), and *p* values are reported. Recruitment source includes Craigslist and UW-Madison Email, with participants coded as 1 if recruited from a given source and 0 otherwise. Missing values ("—") indicate that logistic regression was not conducted for that time point. *p* values in bold indicate statistical significance (*p* < .05). The not estimable confidence interval for recruitment source predicting microbiome WGS quality was related to zero cell count

**Table S11***.* Demographics and Baseline Depression as Predictors of Post-Intervention Questionnaires, Behavioral, and Biological Measure Completion and Quality

|  |  |  |  | **Questionnaire** | | | **Behavioral** | | | **Biological** | | |
| --- | --- | --- | --- | --- | --- | --- | --- | --- | --- | --- | --- | --- |
|  |  |  | *n* | *Odds Ratios* | *CI* | *p* | *Odds Ratios* | *CI* | *p* | *Odds Ratios* | *CI* | *p* |
| **Completion** | | |  |  |  |  |  |  |  |  |  |  |
| **Model 1** | | |  |  |  |  |  |  |  |  |  |  |
|  | Age | | 1,098 | 1.03 | 1.02 – 1.05 | **<.001** | 1.02 | 1.01 – 1.04 | **.003** | 1.03 | 1.01 – 1.04 | **<.001** |
|  | Gender | | 1,098 |  |  |  |  |  |  |  |  |  |
|  |  | *Man* |  | 1.00 | 0.63 – 1.62 | .98 | 0.82 | 0.55 – 1.26 | .35 | 1.05 | 0.74 – 1.49 | .80 |
|  |  | *Other* |  | 0.38 | 0.17 – 0.92 | **.02** | 0.45 | 0.20 – 1.07 | .06 | 0.49 | 0.24 – 1.05 | .06 |
|  | Race/Ethnicity | | 1,098 |  |  |  |  |  |  |  |  |  |
|  |  | *Non-Hispanic White* |  | 1.02 | 0.67 – 1.53 | .93 | 1.14 | 0.78 – 1.65 | .50 | 0.83 | 0.61 – 1.13 | .25 |
|  | Income | | 1,098 |  |  |  |  |  |  |  |  |  |
|  |  | *Above median* |  | 0.92 | 0.61 – 1.39 | .68 | 0.98 | 0.67 – 1.45 | .93 | 1.06 | 0.78 – 1.44 | .73 |
|  | Education | | 1,098 |  |  |  |  |  |  |  |  |  |
|  |  | College Graduate |  | 1.67 | 1.13 – 2.48 | **.01** | 1.56 | 1.08 – 2.23 | **.02** | 1.58 | 1.18 – 2.11 | **.002** |
| **Model 2** | | |  |  |  |  |  |  |  |  |  |  |
|  | PHQ-8 | | 1,098 | 0.81 | 0.59 – 1.13 | .21 | 0.86 | 0.64 – 1.16 | .32 | 0.84 | 0.66 – 1.07 | .16 |
| **Quality** | | |  |  |  |  |  |  |  |  |  |  |
| **Model 1** | | |  |  |  |  |  |  |  |  |  |  |
|  | Age | | 977 | 1.06 | 1.00 – 1.13 | .08 | - | - | - | - | - | - |
|  | Gender | | 977 |  |  |  |  |  |  |  |  |  |
|  |  | *Man* |  | 0.31 | 0.09 – 1.14 | .07 | - | - | - | - | - | - |
|  |  | *Other* |  | 1.86 × 10^7^ | 0.00 – NA | 1.00 | - | - | - | - | - | - |
|  | Race/Ethnicity | | 977 |  |  |  |  |  |  |  |  |  |
|  |  | *Non-Hispanic White* |  | 8.86 | 2.21 – 59.09 | **.01** | - | - | - | - | - | - |
|  | Income | | 977 |  |  |  |  |  |  |  |  |  |
|  |  | *Above median* |  | 3.04 × 10^7^ | 0.00 – NA | .99 | - | - | - | - | - | - |
|  | Education | | 977 |  |  |  |  |  |  |  |  |  |
|  |  | College Graduate |  | 1.09 | 0.31 – 3.97 | .89 | - | - | - | - | - | - |
| **Model 2** | | |  |  |  |  |  |  |  |  |  |  |
|  | PHQ-8 | | 977 | 1.08 | 0.40 – 3.19 | .89 | - | - | - | - | - | - |

*Note.* *n* = 1,098 for completion analyses (completers with demographic information provided); *n* = 977 for quality analyses (questionnaire completers with demographic information provided). OR = odds ratio; CI = 95% confidence interval. Model 1 includes demographic covariates; Model 2 adds baseline depression (PHQ-8), controlling for all demographics as covariates. Reference groups: woman (gender), racial and ethnic minority (race/ethnicity), ≤ median household (income), and < college degree (education). Dashes (—) insufficient variance to predict quality due to high levels of behavioral and biological data quality post-intervention. ORs > 1 indicate higher odds of task completion or high-quality data on one or more measures post-intervention (week 4 and/or 17-week follow-up). *p* < .05. Significant values are bolded. The not estimable confidence interval and very large odds ratio for other gender and above median income in Model 1 predicting quality were related to zero cell counts. Significance tests for all other model coefficients were the same with and without these predictors included.

**Table S12.** Demographics and Baseline PHQ-8 Score as Predictors for Completion of Questionnaire, Video Tasks, Mnemonic Similarity Task, and Biological Measures.

|  |  |  | **Baseline** | | | **Week 1** | | | **Week 2** | | | **Week 3** | | | **Post-treatment** | | | **17-week follow-up** | | |
| --- | --- | --- | --- | --- | --- | --- | --- | --- | --- | --- | --- | --- | --- | --- | --- | --- | --- | --- | --- | --- |
|  |  |  | *Odds Ratios* | *CI* | *p* | *Odds Ratios* | *CI* | *p* | *Odds Ratios* | *CI* | *p* | *Odds Ratios* | *CI* | *p* | *Odds Ratios* | *CI* | *p* | *Odds Ratios* | *CI* | *p* |
| **Questionnaire** | | | | | | | | | | | | | | | | | | | | |
| **Model 1** | | |  |  |  |  |  |  |  |  |  |  |  |  |  |  |  |  |  |  |
|  | Age | | – | – | – | 1.01 | 0.99 – 1.03 | .30 | 1.02 | 1.00 – 1.03 | **.02** | 1.02 | 1.01 – 1.04 | **.001** | 1.02 | 1.01 – 1.04 | **.002** | 1.02 | 1.01 – 1.04 | **.001** |
|  | Gender | |  |  |  |  |  |  |  |  |  |  |  |  |  |  |  |  |  |  |
|  |  | *Man* | – | – | – | 0.96 | 0.62 – 1.55 | .88 | 0.76 | 0.53 – 1.11 | .15 | 0.89 | 0.62 – 1.29 | .54 | 1.11 | 0.73 – 1.75 | .64 | 0.94 | 0.65 – 1.37 | .73 |
|  |  | *Other* | – | – | – | 0.48 | 0.21 – 1.25 | .10 | 0.61 | 0.28 – 1.43 | .23 | 0.87 | 0.39 – 2.12 | .74 | 0.51 | 0.23 – 1.22 | .11 | 0.41 | 0.20 – 0.90 | **.02** |
|  | Race [Non-Latinx White] | | – | – | – | 1.13 | 0.75 – 1.69 | .55 | 1.51 | 1.08 – 2.09 | **.02** | 1.28 | 0.93 – 1.77 | .13 | 1.32 | 0.91 – 1.89 | .14 | 0.67 | 0.47 – 0.95 | **.03** |
|  | Income [Above median] | | – | – | – | 1.21 | 0.80 – 1.87 | .38 | 0.94 | 0.67 – 1.32 | .71 | 1.12 | 0.80 – 1.57 | .52 | 1.12 | 0.76 – 1.65 | .57 | 1.00 | 0.72 – 1.40 | 1.00 |
|  | Education [College Graduate] | | – | – | – | 1.48 | 1.00 – 2.18 | **.05** | 1.22 | 0.88 – 1.69 | .23 | 1.37 | 1.00 – 1.87 | .05 | 1.38 | 0.96 – 1.97 | .08 | 1.64 | 1.20 – 2.26 | **.002** |
| **Model 2** | | |  |  |  |  |  |  |  |  |  |  |  |  |  |  |  |  |  |  |
|  | Baseline PHQ-8 | | – | – | – | 1.09 | 0.79 – 1.53 | .60 | 0.96 | 0.73 – 1.26 | .75 | 1.13 | 0.86 – 1.47 | .38 | 0.86 | 0.64 – 1.15 | .30 | 0.87 | 0.67 – 1.13 | .29 |
| **Stimulus Elicited Affect Task** | | | | | | | | | | | | | | | | | | | | |
| **Model 1** | | |  |  |  |  |  |  |  |  |  |  |  |  |  |  |  |  |  |  |
|  | Age | | 0.98 | 0.96 – 0.99 | **.01** | – | – | – | – | – | – | – | – | – | 1.01 | 1.00 – 1.02 | .23 | 1.01 | 1.00 – 1.02 | .27 |
|  | Gender | |  |  |  |  |  |  |  |  |  |  |  |  |  |  |  |  |  |  |
|  |  | *Man* | 0.68 | 0.44 – 1.07 | .09 | – | – | – | – | – | – | – | – | – | 0.91 | 0.66 – 1.27 | .58 | 1.04 | 0.76 – 1.42 | .83 |
|  |  | *Other* | 2.53 | 0.52 – 45.66 | .37 | – | – | – | – | – | – | – | – | – | 0.69 | 0.33 – 1.53 | .34 | 0.70 | 0.34 – 1.50 | .35 |
|  | Race [Non-Latinx White] | | 1.23 | 0.79 – 1.88 | .35 | – | – | – | – | – | – | – | – | – | 1.06 | 0.79 – 1.42 | .71 | 1.00 | 0.75 – 1.32 | 1.00 |
|  | Income [Above median] | | 0.96 | 0.62 – 1.51 | .67 | – | – | – | – | – | – | – | – | – | 0.96 | 0.71 – 1.29 | .76 | 1.04 | 0.79 – 1.38 | .78 |
|  | Education [College Graduate] | | 1.45 | 0.95 – 2.20 | **.08** | – | – | – | – | – | – | – | – | – | 1.47 | 1.11 – 1.95 | **.01** | 1.51 | 1.16 – 1.98 | **.002** |
| **Model 2** | | |  |  |  |  |  |  |  |  |  |  |  |  |  |  |  |  |  |  |
|  | Baseline PHQ-8 | | 0.82 | 0.59 – 1.15 | .24 | – | – | – | – | – | – | – | – | – | 0.77 | 0.61 – 0.97 | **.03** | 0.86 | 0.69 – 1.07 | .17 |
| **Video Log Task** | | | | | | | | | | | | | | | | | | | | |
| **Model 1** | | |  |  |  |  |  |  |  |  |  |  |  |  |  |  |  |  |  |  |
|  | Age | | 0.98 | 0.96 – 0.99 | **.01** | 1.00 | 0.98 – 1.01 | .58 | 1.01 | 1.00 – 1.02 | .08 | 1.00 | 0.99 – 1.01 | .63 | 1.01 | 1.00 – 1.02 | .23 | 1.01 | 1.00 – 1.02 | .27 |
|  | Gender | |  |  |  |  |  |  |  |  |  |  |  |  |  |  |  |  |  |  |
|  |  | *Man* | 0.68 | 0.44 – 1.07 | .09 | 0.93 | 0.67 – 1.31 | .67 | 0.69 | 0.51 – 0.94 | **.02** | 0.78 | 0.58 – 1.06 | .11 | 0.91 | 0.66 – 1.27 | .58 | 1.04 | 0.76 – 1.42 | .83 |
|  |  | *Other* | 2.53 | 0.52 – 45.66 | .37 | 0.68 | 0.32 – 1.55 | .34 | 0.76 | 0.37 – 1.64 | .47 | 0.98 | 0.46 – 2.20 | .95 | 0.69 | 0.33 – 1.53 | .34 | 0.70 | 0.34 – 1.50 | .35 |
|  | Race [Non-Latinx White] | | 1.23 | 0.79 – 1.88 | .35 | 1.56 | 1.16 – 2.10 | **.003** | 1.34 | 1.02 – 1.77 | **.04** | 1.27 | 0.96 – 1.68 | .09 | 1.06 | 0.79 – 1.42 | .71 | 1.00 | 0.75 – 1.32 | 1.00 |
|  | Income [Above median] | | 0.96 | 0.62 – 1.51 | .86 | 1.04 | 0.77 – 1.42 | .80 | 1.16 | 0.87 – 1.54 | .31 | 0.99 | 0.75 – 1.31 | .93 | 0.96 | 0.71 – 1.29 | .76 | 1.04 | 0.79 – 1.38 | .78 |
|  | Education [College Graduate] | | 1.45 | 0.95 – 2.20 | .08 | 1.60 | 1.20 – 2.15 | **.001** | 1.27 | 0.97 – 1.67 | .08 | 1.20 | 0.92 – 1.57 | .18 | 1.47 | 1.11 – 1.54 | **.01** | 1.51 | 1.16 – 1.98 | **.002** |
| **Model 2** | | |  |  |  |  |  |  |  |  |  |  |  |  |  |  |  |  |  |  |
|  | Baseline PHQ-8 | | 0.82 | 0.59 – 1.15 | .24 | 0.91 | 0.72 – 1.16 | .45 | 0.84 | 0.67 – 1.05 | .13 | 0.98 | 0.78 – 1.23 | .86 | 0.77 | 0.61 – 0.97 | **.03** | 0.86 | 0.69 – 1.07 | .17 |
| **Mnemonic Similarity Task** | | | | | | | | | | | | | | | | | | | | |
| **Model 1** | | |  |  |  |  |  |  |  |  |  |  |  |  |  |  |  |  |  |  |
|  | Age | | 0.98 | 0.96 – 1.01 | .26 | – | – | – | – | – | – | – | – | – | 1.01 | 1.00 – 1.02 | .12 | 1.02 | 1.00 – 1.03 | **.01** |
|  | Gender | |  |  |  |  |  |  |  |  |  |  |  |  |  |  |  |  |  |  |
|  |  | *Man* | 0.33 | 0.16 – 0.69 | **.003** | – | – | – | – | – | – | – | – | – | 0.85 | 0.60 – 1.22 | .38 | 0.92 | 0.66 – 1.30 | .64 |
|  |  | *Other* | 808160.72 | 0.00 – 1.12 x 10^100^ | .98 | – | – | – | – | – | – | – | – | – | 0.72 | 0.33 – 1.70 | .43 | 0.59 | 0.28 – 1.29 | .17 |
|  | Race [Non-Latinx White] | | 1.79 | 0.85 – 3.70 | .12 | – | – | – | – | – | – | – | – | – | 1.36 | 0.99 – 1.86 | .05 | 0.92 | 0.68 – 1.24 | .58 |
|  | Income [Above median] | | 0.95 | 0.44 – 2.19 | .90 | – | – | – | – | – | – | – | – | – | 1.23 | 0.88 – 1.72 | .23 | 1.03 | 0.76 – 1.40 | .84 |
|  | Education [College Graduate] | | 1.87 | 0.89 – 4.05 | .10 | – | – | – | – | – | – | – | – | – | 1.61 | 1.19 – 2.19 | **.002** | 1.63 | 1.22 – 2.17 | **.001** |
| **Model 2** | | |  |  |  |  |  |  |  |  |  |  |  |  |  |  |  |  |  |  |
|  | Baseline PHQ-8 | | 1.31 | 0.70 – 2.54 | .41 | – | – | – | – | – | – | – | – | – | 0.95 | 0.73 – 1.22 | .67 | 0.96 | 0.75 – 1.22 | .71 |
| **Dried Blood Spot Sample** | | | | | | | | | | | | | | | | | | | | |
| **Model 1** | | |  |  |  |  |  |  |  |  |  |  |  |  |  |  |  |  |  |  |
|  | Age | | 1.05 | 0.99 – 1.12 | .12 | – | – | – | – | – | – | – | – | – | – | – | – | 1.03 | 1.02 – 1.04 | **<.001** |
|  | Gender | |  |  |  |  |  |  |  |  |  |  |  |  |  |  |  |  |  |  |
|  |  | *Man* | 1.19 | 0.29 – 7.97 | .83 | – | – | – | – | – | – | – | – | – | – | – | – | 1.09 | 0.77 – 1.54 | . 64 |
|  |  | *Other* | 1.82 x 10^6^ | 0.00 – NA | .99 | – | – | – | – | – | – | – | – | – | – | – | – | 0.53 | 0.25 – 1.11 | .09 |
|  | Race [Non-Latinx White] | | 1.25 | 0.31 – 4.47 | .73 | – | – | – | – | – | – | – | – | – | – | – | – | 0.88 | 0.64 – 1.19 | .40 |
|  | Income [Above median] | | 4.76 | 0.88 – 88.42 | .14 | – | – | – | – | – | – | – | – | – | – | – | – | 1.09 | 0.80 – 1.48 | .59 |
|  | Education [College Graduate] | | 1.23 | 0.33 – 4.54 | .75 | – | – | – | – | – | – | – | – | – | – | – | – | 1.51 | 1.13 – 2.02 | **.01** |
| **Model 2** | | |  |  |  |  |  |  |  |  |  |  |  |  |  |  |  |  |  |  |
|  | Baseline PHQ-8 | | 0.65 | 0.24 – 1.88 | .41 | – | – | – | – | – | – | – | – | – | – | – | – | 0.86 | 0.68 – 1.09 | .22 |
| **Microbiome Sample** | | | | | | | | | | | | | | | | | | | | |
| **Model 1** | | |  |  |  |  |  |  |  |  |  |  |  |  |  |  |  |  |  |  |
|  | Age | | 0.95 | 0.85 – 1.04 | .25 | – | – | – | – | – | – | – | – | – | – | – | – | 1.03 | 1.01 – 1.04 | **<.001** |
|  | Gender | |  |  |  |  |  |  |  |  |  |  |  |  |  |  |  |  |  |  |
|  |  | *Man* | 2.23 x 10^7^ | 0.00 – NA | 1.00 | – | – | – | – | – | – | – | – | – | – | – | – | 1.05 | 0.75 – 1.50 | .76 |
|  |  | *Other* | 1.21 x 10^7^ | 0.00 – NA | 1.00 | – | – | – | – | – | – | – | – | – | – | – | – | 0.50 | 0.24 – 1.07 | .07 |
|  | Race [Non-Latinx White] | | 1.42 | 0.06 – 15.68 | .78 | – | – | – | – | – | – | – | – | – | – | – | – | 0.80 | 0.59 – 1.09 | .16 |
|  | Income [Above median] | | 1.04 | 0.08 – 23.98 | .98 | – | – | – | – | – | – | – | – | – | – | – | – | 1.04 | 0.77 – 1.41 | .80 |
|  | Education [College Graduate] | | 0.69 | 0.03 – 8.29 | .78 | – | – | – | – | – | – | – | – | – | – | – | – | 1.65 | 1.23 – 2.20 | **.001** |
| **Model 2** | | |  |  |  |  |  |  |  |  |  |  |  |  |  |  |  |  |  |  |
|  | Baseline PHQ-8 | | 0.81 | 0.14 – 6.77 | .83 | – | – | – | – | – | – | – | – | – | – | – | – | 0.83 | 0.65 – 1.05 | .13 |

*Note.* Results from logistic regression models predicting completion of various tasks and biological sample collection based on baseline PHQ-8 scores. Odds ratios (OR), 95% confidence intervals (CI), and *p* values are reported. Completion of each task was coded as a binary outcome (1 = completed, 0 = not completed). Missing values ("–") indicate that logistic regression was not conducted for that time point. *p* values in bold indicate statistical significance (*p* < .05). The not estimable confidence interval and very large odds were related to zero cell counts. Significance tests for all other model coefficients were the same with and without these predictors included.

**Table S13.** Demographics and PHQ-8 as Predictors of Questionnaire, Video Tasks and Mnemonic Similarity Task Data Quality

|  |  |  | **Baseline** | | | **Week 1** | | | **Week 2** | | | **Week 3** | | | **Post-treatment** | | | **17-week follow-up** | | |
| --- | --- | --- | --- | --- | --- | --- | --- | --- | --- | --- | --- | --- | --- | --- | --- | --- | --- | --- | --- | --- |
|  |  |  | *Odds Ratios* | *CI* | *P* | *Odds Ratios* | *CI* | *P* | *Odds Ratios* | *CI* | *P* | *Odds Ratios* | *CI* | *P* | *Odds Ratios* | *CI* | *P* | *Odds Ratios* | *CI* | *P* |
| **Questionnaire** | | | | | | | | | | | | | | | | | | | | |
| **Model 1** | | |  |  |  |  |  |  |  |  |  |  |  |  |  |  |  |  |  |  |
|  | Age | | – | – | – | 1.00 | 0.96 – 1.05 | .99 | 0.99 | 0.95 – 1.04 | .68 | 1.00 | 0.95 – 1.04 | .84 | 1.03 | 0.99 – 1.08 | .20 | 1.06 | 1.02 – 1.12 | **.01** |
|  | Gender | |  |  |  |  |  |  |  |  |  |  |  |  |  |  |  |  |  |  |
|  |  | *Man* | – | – | – | 1.11 | 0.33 – 5.04 | .88 | 1.61 | 0.42 – 10.48 | .54 | 0.81 | 0.23 – 3.72 | .76 | 0.56 | 0.19 – 1.87 | .31 | 0.79 | 0.29 – 2.54 | .67 |
|  |  | *Other* | – | – | – | 1.37 x 10^6^ | 0.00 – NA | .99 | 1.72 x 10^6^ | 0.00 – NA | .99 | 0.33 | 0.05 – 6.43 | .32 | 1.64 x 10^6^ | 0.00 – NA | .99 | 3.44 x 10^6^ | 0.00 – NA | .99 |
|  | Race [Non-Latinx White] | | – | – | – | 1.61 | 0.50 – 4.98 | .41 | 1.43 | 0.42 – 4.41 | .54 | 5.52 | 1.74 – 20.86 | **.01** | 3.33 | 1.12 – 11.11 | **.04** | 3.83 | 1.47 – 11.14 | **.01** |
|  | Income [Above median] | | – | – | – | 3.76 | 0.70 – 69.78 | .21 | 1.36 | 0.38 – 6.38 | .65 | 1.68 | 0.48 – 7.75 | .45 | 5.10 | 0.96 – 94.30 | .12 | 2.61 | 0.84 – 11.49 | .14 |
|  | Education [College Graduate] | | – | – | – | 14.34 | 2.74 – 264.15 | **.01** | 2.15 | 0.67 – 7.55 | .21 | 0.93 | 0.28 – 2.94 | .89 | 3.91 | 1.17 – 17.69 | **.04** | 1.39 | 0.54 – 3.65 | .49 |
| **Model 2** | | |  |  |  |  |  |  |  |  |  |  |  |  |  |  |  |  |  |  |
|  | Baseline PHQ-8 | | – | – | – | 1.17 | 0.48 – 3.12 | .74 | 1.17 | 0.48 – 3.15 | .74 | 2.32 | 0.84 – 7.44 | .13 | 1.35 | 0.56 – 3.55 | .53 | 1.23 | 0.57 – 2.87 | .61 |
| **Stimulus Elicited Affect Task** | | | | | | | | | | | | | | | | | | | | |
| **Model 1** | | |  |  |  |  |  |  |  |  |  |  |  |  |  |  |  |  |  |  |
|  | Age | | 0.99 | 0.97 – 1.01 | .47 | – | – | – | – | – | – | – | – | – | 1.00 | 0.98 – 1.02 | .80 | 1.00 | 0.97 – 1.02 | .74 |
|  | Gender | |  |  |  |  |  |  |  |  |  |  |  |  |  |  |  |  |  |  |
|  |  | *Man* | 0.58 | 0.32 – 1.09 | .08 | – | – | – | – | – | – | – | – | – | 0.64 | 0.39 – 1.11 | .10 | 0.63 | 0.35 – 1.18 | .14 |
|  |  | *Other* | 0.43 | 0.14 – 1.92 | .20 | – | – | – | – | – | – | – | – | – | 0.91 | 0.24 – 5.89 | .90 | 0.41 | 0.12 – 1.86 | .18 |
|  | Race [Non-Latinx White] | | 1.24 | 0.69 – 2.19 | .46 | – | – | – | – | – | – | – | – | – | 1.71 | 1.05 – 2.77 | **.03** | 2.02 | 1.17 – 3.48 | **.01** |
|  | Income [Above median] | | 0.69 | 0.39 – 1.25 | .21 | – | – | – | – | – | – | – | – | – | 1.16 | 0.69 – 1.99 | .59 | 0.93 | 0.53 – 1.70 | .82 |
|  | Education [College Graduate] | | 1.69 | 0.97 – 2.99 | .07 | – | – | – | – | – | – | – | – | – | 1.87 | 1.16 – 3.04 | **.01** | 2.06 | 1.20 – 3.59 | **.01** |
| **Model 2** | | |  |  |  |  |  |  |  |  |  |  |  |  |  |  |  |  |  |  |
|  | Baseline PHQ-8 | | 0.87 | 0.55 – 1.40 | .56 | – | – | – | – | – | – | – | – | – | 0.96 | 0.65 – 1.43 | .82 | 0.93 | 0.59 – 1.48 | .75 |
| **Video Log Task** | | | | | | | | | | | | | | | | | | | | |
| **Model 1** | | |  |  |  |  |  |  |  |  |  |  |  |  |  |  |  |  |  |  |
|  | Age | | 0.99 | 0.97 – 1.01 | .37 | 1.01 | 0.99 – 1.03 | .43 | 1.02 | 1.00 – 1.04 | .09 | 1.00 | 0.98 – 1.01 | .60 | 1.02 | 1.00 – 1.03 | .07 | 1.01 | 0.99 – 1.02 | .52 |
|  | Gender | |  |  |  |  |  |  |  |  |  |  |  |  |  |  |  |  |  |  |
|  |  | *Man* | 0.56 | 0.35 – 0.91 | **.02** | 0.59 | 0.37 – 0.96 | **.03** | 0.63 | 0.38 – 1.06 | .07 | 0.83 | 0.51 – 1.39 | .47 | 0.95 | 0.60 – 1.54 | .82 | 0.48 | 0.31 – 0.76 | **.002** |
|  |  | *Other* | 0.76 | 0.25 – 3.29 | .67 | 1.20 | 0.33 – 7.72 | .81 | 0.31 | 0.12 – 0.93 | **.03** | 0.70 | 0.25 – 2.49 | .53 | 0.66 | 0.25 – 2.09 | .44 | 0.83 | 0.26 – 3.68 | .78 |
|  | Race [Non-Latinx White] | | 0.92 | 0.56 – 1.46 | .73 | 0.92 | 0.55 – 1.47 | .72 | 1.38 | 0.86 – 2.20 | .18 | 1.29 | 0.82 – 2.01 | .26 | 1.36 | 0.90 – 2.03 | .14 | 1.49 | 0.96 – 2.28 | .07 |
|  | Income [Above median] | | 0.96 | 0.60 – 1.57 | .88 | 1.29 | 0.80 – 2.12 | .31 | 1.03 | 0.64 – 1.67 | .91 | 1.36 | 0.86 – 2.17 | .19 | 1.48 | 0.97 – 2.33 | .08 | 1.35 | 0.86 – 2.15 | .20 |
|  | Education [College Graduate] | | 2.00 | 1.28 – 3.14 | **.002** | 1.86 | 1.19 – 2.91 | **.01** | 1.53 | 0.97 – 2.40 | .07 | 0.93 | 0.60 – 1.42 | .73 | 1.38 | 0.93 – 2.05 | .11 | 1.42 | 0.93 – 2.17 | .10 |
| **Model 2** | | |  |  |  |  |  |  |  |  |  |  |  |  |  |  |  |  |  |  |
|  | Baseline PHQ-8 | | 0.93 | 0.64 – 1.36 | .71 | 1.12 | 0.77 – 1.65 | .57 | 0.80 | 0.55 – 1.16 | .23 | 0.97 | 0.69 – 1.39 | .88 | 0.79 | 0.57 – 1.10 | .16 | 1.09 | 0.77 – 1.57 | .63 |
| **Mnemonic Similarity Task** | | | | | | | | | | | | | | | | | | | | |
| **Model 1** | | |  |  |  |  |  |  |  |  |  |  |  |  |  |  |  |  |  |  |
|  | Age | | 0.98 | 0.96 – 1.00 | .07 | – | – | – | – | – | – | – | – | – | 1.01 | 0.99 – 1.04 | .28 | 1.00 | 0.98 – 1.03 | .76 |
|  | Gender | |  |  |  |  |  |  |  |  |  |  |  |  |  |  |  |  |  |  |
|  |  | *Man* | 0.86 | 0.48 – 1.62 | .62 | – | – | – | – | – | – | – | – | – | 0.75 | 0.40 – 1.46 | .37 | 0.56 | 0.30 – 1.10 | .08 |
|  |  | *Other* | 2.25 x 10^6^ | 0.00 – 1.21 x 10^98^ | .98 | – | – | – | – | – | – | – | – | – | 3.06 x 10^6^ | 0.00 – NA | .99 | 2.33×10^6^ | 0.00 – NA | .99 |
|  | Race [Non-Latinx White] | | 1.50 | 0.86 – 2.56 | .14 | – | – | – | – | – | – | – | – | – | 1.10 | 0.59 – 1.99 | .75 | 1.31 | 0.68 – 2.44 | .40 |
|  | Income [Above median] | | 0.95 | 0.55 – 1.69 | .86 | – | – | – | – | – | – | – | – | – | 1.59 | 0.86 – 3.12 | .15 | 1.08 | 0.57 – 2.13 | .82 |
|  | Education [College Graduate] | | 0.94 | 0.54 – 1.61 | .81 | – | – | – | – | – | – | – | – | – | 1.11 | 0.62 – 1.98 | .72 | 1.27 | 0.68 – 2.37 | .45 |
| **Model 2** | | |  |  |  |  |  |  |  |  |  |  |  |  |  |  |  |  |  |  |
|  | Baseline PHQ-8 | | 1.01 | 0.65 – 1.60 | .96 | – | – | – | – | – | – | – | – | – | 0.78 | 0.50 – 1.25 | .29 | 0.55 | 0.35 – 0.89 | **.01** |
| **Dried Blood Spot Sample – At least 1 analysis** | | | | | | | | | | | | | | | | | | | | |
| **Model 1** | | |  |  |  |  |  |  |  |  |  |  |  |  |  |  |  |  |  |  |
|  | Age | | – | – | – | – | – | – | – | – | – | – | – | – | – | – | – | 0.97 | 0.92 – 1.03 | .31 |
|  | Gender | |  |  |  |  |  |  |  |  |  |  |  |  |  |  |  |  |  |  |
|  |  | *Man* | – | – | – | – | – | – | – | – | – | – | – | – | – | – | – | 0.35 | 0.09 – 1.43 | .12 |
|  |  | *Other* | – | – | – | – | – | – | – | – | – | – | – | – | – | – | – | 7.49 x 10^5^ | 0.00 – NA | .99 |
|  | Race [Non-Latinx White] | | – | – | – | – | – | – | – | – | – | – | – | – | – | – | – | 2.08 | 0.50 – 8.17 | .29 |
|  | Income [Above median] | | – | – | – | – | – | – | – | – | – | – | – | – | – | – | – | 1.21 | 0.29 – 6.06 | .80 |
|  | Education [College Graduate] | | – | – | – | – | – | – | – | – | – | – | – | – | – | – | – | 0.63 | 0.12 – 2.60 | .53 |
| **Model 2** | | |  |  |  |  |  |  |  |  |  |  |  |  |  |  |  |  |  |  |
|  | Baseline PHQ-8 | | – | – | – | – | – | – | – | – | – | – | – | – | – | – | – | 0.56 | 0.21 – 1.68 | .28 |
| **Dried Blood Spot Sample – All Analyses** | | | | | | | | | | | | | | | | | | | | |
| **Model 1** | | |  |  |  |  |  |  |  |  |  |  |  |  |  |  |  |  |  |  |
|  | Age | | 1.01 | 0.99 – 1.04 | .29 | – | – | – | – | – | – | – | – | – | – | – | – | 1.00 | 0.98 – 1.03 | .98 |
|  | Gender | |  |  |  |  |  |  |  |  |  |  |  |  |  |  |  |  |  |  |
|  |  | *Man* | 0.60 | 0.31 – 1.23 | .15 | – | – | – | – | – | – | – | – | – | – | – | – | 0.49 | 0.27 – 0.93 | **.03** |
|  |  | *Other* | 2.32 x 10^6^ | 0.00 – NA | .99 | – | – | – | – | – | – | – | – | – | – | – | – | 2.21 x 10^6^ | 0.00 – NA | .99 |
|  | Race [Non-Latinx White] | | 0.99 | 0.48 – 1.94 | .98 | – | – | – | – | – | – | – | – | – | – | – | – | 1.37 | 0.72 – 2.53 | .32 |
|  | Income [Above median] | | 1.18 | 0.60 – 2.44 | .65 | – | – | – | – | – | – | – | – | – | – | – | – | 1.17 | 0.61 – 2.36 | .65 |
|  | Education [College Graduate] | | 1.19 | 0.61 – 2.30 | .61 | – | – | – | – | – | – | – | – | – | – | – | – | 1.53 | 0.82 – 2.85 | .18 |
| **Model 2** | | |  |  |  |  |  |  |  |  |  |  |  |  |  |  |  |  |  |  |
|  | Baseline PHQ-8 | | 0.77 | 0.46 – 1.32 | .33 | – | – | – | – | – | – | – | – | – | – | – | – | 1.28 | 0.77 – 2.20 | .36 |

*Note.* Results from logistic regression models predicting quality of various tasks and biological sample collection based on baseline PHQ-8 scores. Odds ratios (OR), 95% confidence intervals (CI), and *p* values are reported. Quality was coded as a binary outcome (1 = good quality, 0 = poor quality), as described in the methods section. Missing values ("–") indicate that logistic regression was not conducted for that time point. *p* values in bold indicate statistical significance (*p* < .05). The not estimable confidence interval and very large odds were related to zero cell counts. Significance tests for all other model coefficients were the same with and without these predictors included.

**References**

1. Babor, T. F., Higgins-Biddle, J. C., Saunders, J. B., & Monteiro, M. G. (2001). The Alcohol Use Disorders Identification Test: Guidelines for use in primary health care. (Second Ed.) Geneva: World Health Organization

2. Berman AH, Bergman H, Palmstierna T, Schlyter F. Evaluation of the Drug Use Disorders Identification Test (DUDIT) in criminal justice and detoxification settings and in a Swedish population sample. Eur Addict Res. 2004;11(1):22-31. doi: 10.1159/000081413

3. Spitzer RL, Kroenke K, Williams JBW, Löwe B. A brief measure for assessing generalized anxiety disorder: the GAD-7. Arch Intern Med. 2006;166(10):1092-1097. doi: 10.1001/archinte.166.10.1092

4. Kroenke K, Strine TW, Spitzer RL, Williams JBW, Berry JT, Mokdad AH. The PHQ-8 as a measure of current depression in the general population. J Affect Disord. 2009;114(1-3):163-173. doi: 10.1016/j.jad.2008.06.026

5. Kroenke K, Spitzer RL, Williams JBW. The PHQ-9: validity of a brief depression severity measure. J Gen Intern Med. 2001;16(9):606-613. doi: 10.1046/j.1525-1497.2001.016009606.x

6. Cohen S, Williamson G. Perceived stress in a probability sample of the United States. In: Spacapan S, Oskamp S, eds. The Social Psychology of Health. Sage Publications; 1988:31-67.

7. Yu L, Buysse DJ, Germain A, et al. Development of short forms from the PROMIS™ sleep disturbance and sleep-related impairment item banks. Behav Sleep Med. 2011;10(1):6-24. doi: 10.1080/15402002.2012.636266

8. Amtmann D, Cook KF, Jensen MP, et al. Development of a PROMIS item bank to measure pain interference. Pain. 2010;150(1):173-182. doi: 10.1016/j.pain.2010.04.025

9. Cella D, Riley W, Stone A, et al. The Patient-Reported Outcomes Measurement Information System (PROMIS) developed and tested its first wave of adult self-reported health outcome item banks: 2005-2008. J Clin Epidemiol. 2010;63(11):1179-1194. doi: 10.1016/j.jclinepi.2010.04.011

10. Juniper EF, O’Byrne PM, Guyatt GH, Ferrie PJ, King DR. Development and validation of a questionnaire to measure asthma control. Eur Respir J. 1999;14(4):902-907. doi: 10.1034/j.1399-3003.1999.14d29.x

11. Clayton S, Karazsia BT. Development and validation of a measure of climate change anxiety. J Environ Psychol. 2020;69:101434. doi: 10.1016/j.jenvp.2020.101434

12. Fresco DM, Moore MT, van Dulmen MHM, et al. Initial psychometric properties of the experiences questionnaire: validation of a self-report measure of decentering. Behav Ther. 2007;38(3):234-246. doi: 10.1016/j.beth.2006.08.003

13. Baer RA, Smith GT, Hopkins J, Krietemeyer J, Toney L. Using self-report assessment methods to explore facets of mindfulness. Assessment. 2006;13(1):27-45. doi: 10.1177/1073191105283504

14. Diener E, Wirtz D, Tov W, et al. New well-being measures: short scales to assess flourishing and positive and negative feelings. Soc Indic Res. 2010;97(2):143-156. doi: 10.1007/s11205-009-9493-y

15. Dweck CS. Mindset: The New Psychology of Success. Random House; 2006.

16. Kral, T. R., Kesebir, P., Redford, L. et al. (2024). Healthy Minds Index: A brief measure of the core dimensions of well-being. PloS one, 19(5), e0299352. doi: 10.1371/journal.pone.0299352

17. Schultz PW. Inclusive with nature: the psychology of human-nature relations. In: Schmuck P, Schultz WP, eds. Psychology of Sustainable Development. Kluwer Academic Publishers; 2002:61-78. doi: 10.1007/978-1-4615-0995-0_4

18. Scheier MF, Carver CS, Bridges MW. Distinguishing optimism from neuroticism (and trait anxiety, self-mastery, and self-esteem): a reevaluation of the Life Orientation Test. J Pers Soc Psychol. 1994;67(6):1063-1078. doi: 10.1037//0022-3514.67.6.1063

19. Cyranowski JM, Zill N, Bode R, et al. Assessing social support, companionship, and distress: NIH Toolbox Adult Social Relationship Scales. Health Psychol. 2013;32(3):293-301. doi: 10.1037/a0028586

20. Salsman JM, Lai JS, Hendrie HC, et al. Assessing psychological well-being: self-report instruments for the NIH Toolbox. Qual Life Res. 2014;23(1):205-215. doi: 10.1007/s11136-013-0452-3

21. Moreno MA, Arseniev-Koehler A, Selkie E. Development and testing of a 3-item screening tool for problematic internet use. J Adolesc Health. 2016;59(3):305-311. doi: 10.1016/j.jpeds.2016.05.067

22. Ford, D. C., Merrick, M. T., Parks, S. E., Breiding, M. J., Gilbert, L. K., Edwards, V. J., ... & Thompson, W. W. (2014). Examination of the factorial structure of adverse childhood experiences and recommendations for three subscale scores. Psychology of Violence, 4(4), 432-444. doi: 10.1037/a0037723

23. Diener E, Emmons RA, Larsen RJ, Griffin S. The Satisfaction with Life Scale. J Pers Assess. 1985;49(1):71-75. doi: 10.1207/s15327752jpa4901_13

24. Lyubomirsky S, Lepper HS. A measure of subjective happiness: preliminary reliability and construct validation. Soc Indic Res. 1999;46(2):137-155. doi: 10.1023/A:1006824100041

25. Ford, D. C., Merrick, M. T., Parks, S. E., Breiding, M. J., Gilbert, L. K., Edwards, V. J., ... & Thompson, W. W. (2014). Examination of the factorial structure of adverse childhood experiences and recommendations for three subscale scores. Psychology of Violence, 4(4), 432-444.

26. John OP, Srivastava S. The Big Five trait taxonomy: history, measurement, and theoretical perspectives. In: Pervin LA, John OP, eds. Handbook of Personality: Theory and Research. 2nd ed. Guilford Press; 1999:102-138.

27. Hunt, C. A., Hoffman, M. A., Mohr, J. J., & Williams, A. L. (2020). Assessing perceived barriers to meditation: the determinants of meditation practice inventory-revised (DMPI-R). Mindfulness, 11, 1139-1149. doi: 10.1007/s12671-020-01308-7

28. Henson P, Wisniewski H, Hollis C, Keshavan M, Torous J. Digital mental health apps and the therapeutic alliance: initial review. BJPsych Open. 2019;5(1):e15. doi: 10.1192/bjo.2018.86

29. Crowne DP, Marlowe D. A new scale of social desirability independent of psychopathology. J Consult Psychol. 1960;24(4):349-354. doi: 10.1037/h0047358

30. Beloborodova, P., Smith, L. M., Riordan, K. M., Simonsson, O., Dottori, L. T., Song, H. Q., Shashko, N. S., Tatar, R., Baldwin, S. A., Bernstein, A., Dunne, J. D., Davidson, R. J., Hirshberg, M. J., Goldberg, S. B. (in press). Prevalence and predictors of self-reported adverse experiences in digital meditation training: 2 randomized controlled trials. JMIR Mental Health. doi: 10.2196/90602

31. First MB, Williams JBW, Karg RS, Spitzer RL. Structured Clinical Interview for DSM-5 Disorders, Clinician Version (SCID-5-CV). American Psychiatric Association Publishing; 2016.

32. Stark, C. E., Noche, J. A., Ebersberger, J. R., Mayer, L., & Stark, S. M. (2023). Optimizing the mnemonic similarity task for efficient, widespread use. Frontiers in Behavioral Neuroscience, 17, 1080366. doi: 10.3389/fnbeh.2023.1080366.33. McDade TW, Williams S, Snodgrass JJ. What a drop can do: dried blood spots as a minimally invasive method for integrating biomarkers into population-based research. Demography. 2007;44(4):899-925. doi:10.1353/dem.2007
