# Supplementary material for: Incorporating Objective Behavioral and Biological Measures in a Large-Scale Decentralized Mobile Health Trial for Depression: Randomized Controlled Trial
Source: JMIR Ment Health. 2026 Oct 9;13:e92589. doi: 10.2196/92589 (PMC13698800; doi:10.2196/92589)
Supplement: Supplementary file 2 [file mental-v13-e92589-s002.docx]

**Consolidated Standards of Reporting Trials of Electronic and Mobile Health Applications and online TeleHealth (CONSORT-EHEALTH) V.1.6.1 checklist**

| **Section / Topic** | **No** | **CONSORT-EHEALTH checklist item** | **How addressed in manuscript** | **Reported on page no.** |
| --- | --- | --- | --- | --- |
| **Title and abstract** | | | |  |
| Title | 1a-i | Identification as a randomized trial in the title; mode of delivery (eg, “web-based”, “mobile”) in title | Title includes “randomized controlled trial” and “mobile.” | 1 |
|  | 1a-ii | Non-web-based components or co-interventions in title | NA – no non-web-based co-interventions; title notes remote behavioral and biological measures. | 1 |
|  | 1a-iii | Primary condition or target group in title | Title includes “Depression” | 1 |
| Structured abstract | 1b-i | Structured summary; key features of intervention and comparator | 3-arm randomized DCT of the Healthy Minds Program (HMP) app, a self-guided DMHI with didactic well-being and meditation content. Comparators (HMP-Psychoed, usual care) not named in abstract. | 1 |
|  | 1b-ii | Level of human involvement in abstract | Abstract includes “Self-guided” and “DCT” | 1 |
|  | 1b-iii | Recruitment (online vs offline), purely web-based vs face-to-face; self-assessed outcomes | Recruited remotely (Craigslist, university emails), all 50 US states; fully remote (questionnaires, clinical interview, self-collected blood/stool, video tasks). | 1–2 |
|  | 1b-iv | Results include use data: N per group, uptake/attrition | N = 1,156 randomized; 84.00% retained; completion and data-quality rates reported. N per group and app-use data not reported but are reported in CONSORT diagram. | 2 |
|  | 1b-v | Conclusions for negative trials | NA – beyond the scope of this paper. | 2 |
| **Introduction** | | | |  |
| Background and objectives | 2a-i | Problem and type of system; stand-alone vs integrated; target population; goals | DCTs and DMHIs lack remote objective (behavioral/biological) measures; HMP is a stand-alone smartphone DMHI tested in an adult sample selected for elevated depressive symptoms. | 2–3 |
|  | 2a-ii | Scientific background, rationale; justify comparator | Background on DCTs, DMHIs, and biomarkers of depression. HMP-Psychoed isolates the contribution of meditation beyond didactic content. | 2–3, 5 |
|  | 2b | Specific objectives or hypotheses | Describe BeWell methodology; secondarily, assess feasibility (completion, data quality, predictors). | 3 |
| **Methods** | | | |  |
| Trial design | 3a | Description of trial design, including allocation ratio | Prospectively registered, 3-arm, longitudinal, randomized DCT; 2:2:1 allocation (HMP-Full, HMP-Psychoed, usual care). | 3–4 |
|  | 3b-i | Important changes to methods after commencement (bug fixes, downtimes, content changes) | Teddy–REDCap technical error assigned some participants extra SEA tasks in weeks 1–3; recruitment-source item added after study start; 1 participant randomized in error and excluded. | 5–7, 10–11 |
| Participants | 4a-i | Eligibility criteria; computer/Internet literacy | Age 18–65, PHQ-9 ≥5, smartphone access (iOS/Android), English proficiency; exclusions listed. | 4 |
|  | 4a-ii | Open vs closed recruitment; web-based vs face-to-face; prevention of multiple identities | Online and offline recruitment (Craigslist, social media, emails, flyers, etc.); fully remote with video clinical interview. Duplicates screened via email matching and Levenshtein distance (n = 5 excluded). | 3–4, 6, 8 |
|  | 4a-iii | Information given during recruitment and consent | REDCap landing page with a video of the PI describing the study and a virtual consent form. | 3 |
|  | 4b-i | Settings/locations; outcomes self-assessed online or otherwise | Participants’ homes across the US. Self-report questionnaires online via REDCap; clinical interview via Webex; self-collected biological samples; video EMA via Teddy app; MST via smartphone. | 3–7 |
|  | 4b-ii | How institutional affiliations were displayed | UW–Madison and Center for Healthy Minds channels used in recruitment; PI video on landing page. | 3 |
| Interventions | 5-i | Names, credentials, affiliations of developers, sponsors, owners | HMP developed by Humin (nonprofit); authors RT, CJD, RJD affiliated (declared). Teddy app built for the study by CF, a Senior Software Engineer | 3, 13, 15 |
|  | 5-ii | History/development process; prior formative evaluations | Prior HMP research cited [3, 30, 31]; Teddy developed in ~1 month. Formative evaluations not reported. | 5, 13 |
|  | 5-iii | Revisions and updating; version/date; content frozen? | Study HMP nearly identical to public version, with modified introductory content and main-page layout. Version number/date not reported. Teddy study version publicly available on GitHub [33]. | 5 |
|  | 5-iv | Quality assurance of information provided | N/A – not directly within current paper scope. | — |
|  | 5-v | Replicability: source code, screenshots, flowcharts | Teddy app open-source [33]; Teddy illustration (Fig 2); study overview (Fig 1). HMP screenshots are not provided since HMP is publicly available. | 4–5, 13 |
|  | 5-vi | Digital preservation: URL, archiving | HMP is free and publicly available; Teddy repository cited (GitHub, ref 33). URLs/archiving not provided in text. | 4–5 |
|  | 5-vii | Access: how participants accessed the app; payment; setting | REDCap emailed download link that linked usage to participant ID; app free; smartphone required. Participants paid up to $265 for assessments (not app use). UC offered public HMP after study. | 4–6 |
|  | 5-viii | Mode of delivery, features/components, theoretical framework, content | Smartphone app. HMP-Full: psychoeducation + >100 guided meditations across 4 pillars (awareness, connection, insight, purpose) [3, 30, 31]. HMP-Psychoed: psychoeducation only. UC: no study intervention. | 4–5 |
|  | 5-ix | Use parameters (intended dose, timing) | Encouraged to use app daily during the 4-week intervention, but not required. | 4 |
|  | 5-x | Level of human involvement | Self-guided; no human support for intervention. Staff (2.5 FTE) supported study procedures; baseline clinical interviews for assessment only. | 4, 6 |
|  | 5-xi | Prompts/reminders used | Automated REDCap email if app not downloaded within 1 week (with instructional video); automated reminders for study activities; birthday cards. | 6, 8 |
|  | 5-xii | Co-interventions (incl. training/support) | All participants received a list of national mental health resources (eg, 988); UC continued usual care. Optional video call help for biological sample collection. | 7, 10 |
| Outcomes | 6a-i | Pre-specified outcomes; online questionnaires validated for online use; CHERRIES | Paper reports feasibility outcomes (recruitment, retention, completion, data quality); clinical outcomes not reported. Standardized questionnaires via REDCap (Table S2) with attention check. Online validation/CHERRIES not reported. | 3, 5–6, 8 |
|  | 6a-ii | How “use” was defined/measured | HMP logged date, time, duration, device, lesson/practice, week, and category. Use outcomes not reported or within scope of this paper. | 6 |
|  | 6a-iii | Qualitative feedback from participants | NA | — |
|  | 6b | Changes to trial outcomes after commencement, with reasons | NA | — |
| Sample size | 7a-i | How sample size was determined; attrition accounted for | NA | — |
|  | 7b | Interim analyses and stopping guidelines | NA | — |
| Randomization: Sequence generation | 8a | Method used to generate random allocation sequence | REDCap randomization module. | 4 |
|  | 8b | Type of randomization; restriction (blocking, block size) | 2:2:1 allocation; blocking/stratification not reported. | 4 |
| Allocation concealment | 9 | Mechanism to implement allocation; concealment | REDCap module, after completion of baseline measures. | 4 |
| Implementation | 10 | Who generated sequence, enrolled, and assigned participants | REDCap module assigned participants after baseline completion; individuals responsible not reported. | 4 |
| Blinding | 11a-i | Who was blinded and who was not | Not reported. | — |
|  | 11a-ii | Informed consent and participant expectations re: intervention of interest | Informed consent described on pg. 3. Participant expectations not assessed or reported. | 3 |
|  | 11b | Similarity of interventions | HMP-Psychoed is HMP with guided meditations removed. | 5 |
| Statistical methods | 12a-i | Methods to compare groups; handling of attrition/missing data (imputation) | Descriptive statistics and binary logistic regressions (ORs, 95% CIs); groups not compared. Completion/quality coded as binary outcomes; no imputation reported; duplicates (n = 5) excluded. | 8 |
|  | 12b | Additional analyses (subgroup, adjusted) | Predictors: recruitment source, demographics, depression severity; demographics as covariates for depression models. | 8 |
| Ethics and informed consent | X26-i | Ethics committee approval | UW–Madison Health Sciences IRB (2021-0991); Declaration of Helsinki. | 8 |
|  | X26-ii | Informed consent procedures (online/offline) | Electronic consent via virtual consent form on REDCap landing page; consent for video/audio recording. | 3, 7–8 |
|  | X26-iii | Safety, security, privacy procedures | HIPAA-compliant REDCap and Webex; video encrypted in transit and on servers; limited staff access; de-identified interview records; mental health resource list. | 3–4, 6–7, 10 |
| **Results** | | | |  |
| Participant flow | 13a | Numbers randomly assigned, receiving treatment, and analyzed per group | 52,052 screened; 1,156 randomized: HMP-Full 462, HMP-Psychoed 463, UC 232 (CONSORT diagram, Fig 3). Analyses pooled across arms. | 9–10 |
|  | 13b-i | Losses/exclusions with reasons; attrition diagram of usage | Losses and reasons in Fig 3; 971 (84.00%) retained; completion by timepoint in Table 1 and Fig S6. | 9–10 |
| Recruitment | 14a-i | Dates of recruitment/follow-up; secular events | Recruitment May 31, 2022–Dec 11, 2023; data collection June 1, 2022–July 15, 2024. Secular events not reported (COVID-19 context discussed). | 2, 4, 8 |
|  | 14b | Why the trial ended or was stopped | NA – not stopped early; data collection completed July 15, 2024. | 4 |
| Baseline data | 15-i | Baseline characteristics per group; digital-divide demographics | Demographics in Table S7: age, gender, race/ethnicity; income and education used as predictors. Computer/eHealth literacy not reported. | 8, 12 |
| Numbers analyzed | 16-i | Denominators per analysis; multiple use thresholds | n/N per measure and timepoint reported | 8–12 |
|  | 16-ii | Intent-to-treat primary analysis | NA – efficacy not analyzed; all randomized participants (excluding errors/duplicates) included. | 8, 10 |
| Outcomes and estimation | 17a-i | Results per group with effect size and precision; use/dose metrics | NA – clinical outcomes not reported. Feasibility outcomes reported with ORs and 95% CIs. Use metrics not reported. | 9–12 |
|  | 17b | Absolute and relative effect sizes for binary outcomes | Percentages and ORs (95% CI) for completion/quality. | 9–12 |
| Ancillary analyses | 18-i | Other analyses (subgroup, adjusted); users-only analyses | Predictors of completion/quality by recruitment source, demographics, and depression severity (Tables 2, S8–S13). No users-only analysis. | 11–12 |
| Harms | 19-i | Harms/unintended effects incl. privacy breaches, technical problems | Harms/privacy breaches did not occur. Technical issues: Teddy–REDCap error (extra SEA tasks); $50 bonus to 61.51% who completed/attempted all measures incl. technical or shipping issues. | 5–7, 9 |
|  | 19-ii | Qualitative feedback/staff observations | No participants requested remote walk-through; switching devices for video EMA was challenging. Participant qualitative feedback not assessed or reported. | 9, 13 |
| **Discussion** | | | |  |
| Principal findings | 22-i | Restate study questions; summarize answers (primary and use outcomes) | Methodology feasible: ≥68% completion, ≥84% analyzable data, 84.00% retention. | 12 |
|  | 22-ii | Unanswered questions; future research | Oversampling underrepresented groups, streamlining video EMA and assessments, studying reasons for non-enrollment. | 13–14 |
| Limitations | 20-i | Limitations: blinding, multiplicity, non-use, consent bias, unexpected events | Video EMA workflow burden, demographic differences in completion, battery length, selection bias. Blinding and multiplicity not discussed. | 13–14 |
| Generalizability | 21-i | Generalizability to other populations and settings | Limited by recruitment sources, 2.22% screen-to-randomization ratio, depression-related non-completion, and country-specific regulations. | 13–14 |
|  | 21-ii | Elements of the RCT that differ from routine application | Required interview and biological samples before randomization likely increased retention; payments/bonus may have aided completion. Routine-use differences not otherwise discussed. | 13–14 |
| **Other information** | | | |  |
| Registration | 23 | Registration number and trial registry | ClinicalTrials.gov NCT05183867. | 2–3 |
| Protocol | 24 | Where the full trial protocol can be accessed | OSF registration cited (ref 23); measures listed on ClinicalTrials.gov. Data and code on OSF. | 3, 5, 14 |
| Funding | 25 | Sources of funding; role of funders | NIH, Hope for Depression Research Foundation, Brain & Behavior Research Foundation, and others; funders had no role. | 3, 14 |
| Competing interests | X27-i | Relation of study team to system evaluated | RT, CJD, RJD affiliated with Humin (HMP developer); RP holds shares in SmartEye AB (owns Affectiva, Affdex software used); other interests declared. | 15 |

Note. Page numbers refer to the published article, NA = not applicable; “Not reported” = applicable but not described in the manuscript. DCT = decentralized clinical trial; DMHI = digital mental health intervention; HMP = Healthy Minds Program; UC = usual care; SEA = stimulus-elicited affect; EMA = ecological momentary assessment; MST = Mnemonic Similarity Task; OR = odds ratio; OSF = Open Science Framework.
